# Designing of the N-ethyl-4-(pyridin-4-yl)benzamide based potent ROCK1 inhibitors using docking, molecular dynamics, and 3D-QSAR

**DOI:** 10.7717/peerj.11951

**Published:** 2021-08-09

**Authors:** Suparna Ghosh, Seketoulie Keretsu, Seung Joo Cho

**Affiliations:** 1 Department of Biomedical Sciences, College of Medicine, Chosun University, Gwangju, South Korea; 2 Department of Cellular and Molecular Medicine, College of Medicine, Chosun University, Gwangju, South Korea

**Keywords:** Rho-associated kinase-1 (ROCK1), Cardio-vascular disease, Molecular docking, Molecular dynamics, MMPBSA, 3D-QSAR, CoMFA, CoMSIA, ADME/Tox

## Abstract

Rho-associated kinase-1 (ROCK1) has been recognized for its pivotal role in heart diseases, different types of malignancy, and many neurological disorders. Hyperactivity of ROCK phosphorylates the protein kinase-C (PKC), which ultimately induces smooth muscle cell contraction in the vascular system. Inhibition of ROCK1 has been shown to be a promising therapy for patients with cardiovascular disease. In this study, we have conducted molecular modeling techniques such as docking, molecular dynamics (MD), and 3-Dimensional structure-activity relationship (3D-QSAR) on a series of N-ethyl-4-(pyridin-4-yl)benzamide-based compounds. Docking and MD showed critical interactions and binding affinities between ROCK1 and its inhibitors. To establish the structure-activity relationship (SAR) of the compounds, 3D-QSAR techniques such as Comparative Molecular Field Analysis (CoMFA) and Comparative Molecular Similarity Indices Analysis (CoMSIA) were used. The CoMFA (*q*^2^ = 0.774, *r*^2^ = 0.965, ONC = 6, and ${r}_{pred}^{2}$ = 0.703) and CoMSIA (*q*^2^ = 0.676, *r*^2^ = 0.949, ONC = 6, and ${r}_{pred}^{2}$ = 0.548) both models have shown reasonable external predictive activity, and contour maps revealed favorable and unfavorable substitutions for chemical group modifications. Based on the contour maps, we have designed forty new compounds, among which, seven compounds exhibited higher predictive activity (pIC_50_). Further, we conducted the MD study, ADME/Tox, and SA score prediction using the seven newly designed compounds. The combination of docking, MD, and 3D-QSAR studies helps to understand the coherence modification of existing molecules. Our study may provide valuable insight into the development of more potent ROCK1 inhibitors.

## Introduction

**Figure 1:**
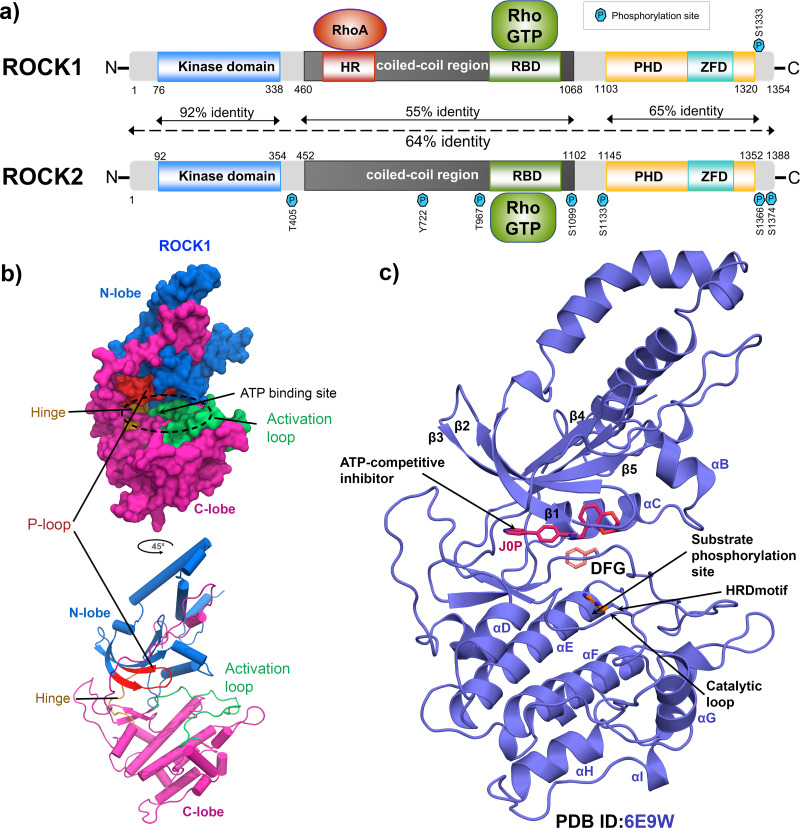
Domain maps and X-ray structure of human ROCK1 protein. (A) ROCK1 and ROCK2 isoforms share 64% overall sequence identity. The highest homology of 92%, was found in the N-terminal kinase domain. Both the isoforms can be activated when binding with the RhoGTP by their Rho-binding domain (RBD). Additionally, ROCK1 consists of an HR domain in its coiled-coil region, which binds to RhoA. The zink finger-like domain (ZFD) is located at the C-terminal pleckstrin homology domain (PHD) in both the isoforms. The heptagonal blue P signs are indicating the different phosphorylation sites. (B) N-lobe, C-lobe, Activation loop, P-loop, ATP binding site, and Hinge loop were shown in different color schemes by surface model and cartoon representation. (C) Global structure of the inhibitor bound ROCK1 showing the ATP pocket, phosphorylation site, catalytic loop, HRD, and DFG motif.

Rho-associated kinase (ROCK) has been studied for its role as a downstream effector of small guanosine triphosphatases (GTPases), RhoA, RhoB, and RhoC (Tang, Campbell & Nithipatikom, 2012; Vigil et al., 2012). It is a member of the serine/threonine AGC kinase family and phosphorylates various downstream targets such as myosin light chain 2 (MLC2), myosin light chain phosphatase 1 (MYPT1), LIM kinase, collapsing response mediator protein-2 (CRMP-2), adducin and calponin. The downstream phosphorylation promotes actin–myosin-mediated smooth muscle cell contraction, actin organization, cell proliferation, and migration (Lock et al., 2012; Shi et al., 2013; Sunamura et al., 2018; Kalaji et al., 2012). ROCK1 and ROCK2 are two isoforms of the ROCK family and consist of 1354 and 1388 amino acids, respectively. ROCK1 and ROCK2 share 65% overall sequence identity and 92% amino acid homology in their kinase domains as shown in Fig. 1A (Judge et al., 2018; Heikkila et al., 2011). Full-length ROCKs contain an N-terminal kinase domain, a coiled-coil linker region, and a Rho-binding domain (RBD). The split pleckstrin homology (PH) domain, containing a zink finger-like cystine-rich tandem repeat domain (ZFD), is located at the C-terminal end and shares 65% sequence identity between the two isoforms. The ROCKs exclusively interact with the switch-1 and switch-2 regions of the active RhoA, RhoB, and RhoC conjugated to GTP by their RBD domains (Hartmann, Ridley & Lutz, 2015). However, ROCK1 and ROCK2 have different binding preferences for membrane lipids. Their subcellular localizations were also found to be distinct. ROCK1 has been reported to have cytosolic localization and is closely associated with cell–cell adhesion, plasma membrane, and centrosomes, while ROCK2 is more abundant in the intercalated disk and Z-disk of striated muscle cells (Lock et al., 2012; Shi et al., 2013; Lisowska et al., 2018). In particular, ROCK1 regulates cell motility integrin *β*1-activated focal adhesion kinase (FAK) signaling during multiple myeloma and lung cancer progression (Amit et al., 2020; Gilkes et al., 2014). Overexpression of ROCK1 is correlated with numerous pathophysiological conditions such as cardiac arrest, cerebral vasospasm, pulmonary hypertension, reperfusion injury, glaucoma, arteriosclerosis, Alzheimer’s disease, and several types of cancer (Jin et al., 2019; Shi et al., 2010; Shao et al., 2008; Hu et al., 2016). Thus, the pharmaceutical industry is focusing on the development of selective and nonselective ATP competitive ROCK inhibitors (Feng et al., 2016). Figures 1B and 1C depicted the global structure of the ROCK1 kinase domain and an ATP competitive Type-1 inhibitor inside the binding pocket (PDB ID: 6E9W). The primary role of this inhibitor is to block the transmission of terminal phosphate from ATP to its corresponding substrate (Koch et al., 2018). These inhibitors encompass a variety of chemical groups, such as pyridine, pyrazole, indazole, amino-furazan, and isoquinoline derivatives, which form critical interactions with the hinge loop at the binding pocket of kinase domain (Feng et al., 2007; Goodman et al., 2007; Abbhi et al., 2017; Schirok et al., 2008; Fang et al., 2010; Gingras et al., 2004).

The computational modeling approach would play a viable role in the discovery of more potent lead compounds as ROCK inhibitors. We have conducted a molecular modeling study of 42 novel N-ethyl-4-(pyridin-4-yl)benzamide-based compounds reported by Hobson et al. (2018), which showed a wide range of inhibitory activity (IC_50_ =0.003 µM - 16 µM) against ROCK1. Compound C08, N-((2,3-Dihydrobenzo[b][1,4]dioxin-5-yl)methyl)-4-(pyridine-4-yl)benzamide which was cocrystallized with ROCK1 (PDB ID: 6E9W), was selected as a representative candidate among the compounds. To investigate the critical binding interaction, we performed molecular docking and MD studies of C08, along with several other compounds in the dataset. In addition, we have calculated the MMPBSA binding-free energy and interaction energy (IE) to evaluate the binding affinity. Based on the average MD pose of C08, we developed the CoMFA and CoMSIA models to establish the structure–activity (SAR) relationship. Finally, we designed 40 new compounds and their activities (pIC_50_) were predicted based on the CoMFA and CoMSIA models. The designed compounds with higher pIC_50_ values were taken for the Absorption Distribution Metabolism Excretion Toxicity (ADME/Tox), and Synthetic Accessibility (SA) score analysis.

## Methodology

### Molecular docking

Molecular docking experiments of the compounds C03, C08, C11, C13, C19, C22, C25, C29, C34, and C37 have been carried out using AutoDockTools (AutoDock 4.2, Scripps Research, CA, USA) as described by earlier studies (Keretsu, Bhujbal & Cho, 2019; Keretsu, Bhujbal & Cho, 2020). The high-resolution crystal structure of ROCK1 (PDB ID:6E9W) was retrieved from the protein databank and used as a receptor in the docking study. Ligands and water molecules were removed from the original structure. The missing loop (residue S249-G253 and residue L372-T279) has been modeled with Modeller-10.1 (University of San Francisco, California, USA) and validated by Ramachandran plot analysis on the PROCHECK (DOE-MBI services, UCLA) server (Fig. S1). The pyridinylbenzamide-based inhibitor, compound C08 in our dataset, is already available in the co-crystallized form. Therefore, C08 was treated as the most selective compound in our dataset. Compound C34 showed the highest inhibitory activity and was designated as the most active compound. Compounds C03, C11, C13, C19, C22, C25, C29, and C37 have different chemical subgroups in their positions R_1_, R_2_, R_3_ and R_4_ and exhibited a diverse range of activities against ROCK1, which were also included in the docking study. C08 was extracted and re-docked into the binding pocket of ROCK1. In brief, the protein was prepared by adding polar hydrogen and Kollman charges. The ligand was prepared by the addition of missing polar and nonpolar hydrogen atoms and assigned Gasteiger charges. To avoid excessive conformational explosions, the number of rotatable bonds was fixed to six. The AutoGrid program was used to define the grid box size of 50  × 50  × 60 points in the X, Y, and Z directions with the grid spacing of 0.375, in such a way that it could conceal the entire ligand, while the grid center was set at X = −65.0, Y = −8.0 and Z = −45, respectively. AutoDock−4.2 was used for the conformational search using the Lamarckian Genetic Algorithm (LGA). The other docking parameters were set to default, and 100 docking runs were executed. The most suitable protein-ligand docking conformation was selected from the lowest binding energy with the lowest RMSD cluster of 100 runs. Polar, nonpolar, and hydrophobic interactions were also considered to evaluate the final protein-ligand binding pose. This docking protocol was repeated for compounds C03, C11, C13, C19, C22, C25, C29, and C37. Finally, the docking analysis was performed using AutoDockTools, PyMOL (Schrodinger, Inc.), and LigPlot+ (EMBL-EBI, Cambridgeshire, UK).

### MD simulation

The MD simulations were run in GROMACS 2019.5 (Lindahl et al., 2020) using CHARMM36 all-atom Force Field (2020). The ligand topology and parameter files with partial charges were generated using the CHARMM General Force Field (CGenFF) (Massachusetts, USA). The protein-ligand docked complex was solvated with TIP3P water in a cubic periodic box model. The minimum distance between the protein and the box walls was maintained at 10 Å in the X, Y, and Z directions. The Na^+^ and Cl^−^ counterions were added adequately to neutralize the system by replacing the solvent molecules. The entire system was subjected to energy minimization using the steepest descent integrator. Thereafter, the system was equilibrated for 200 ps in the constant volume (NVT) ensemble to achieve the temperature of 300 K, followed by 400 ps in the constant pressure (NPT) ensemble to achieve 1 bar of pressure. The protein backbone was kept restrained during the NVT and NPT simulations. The particle mesh Ewald (PME) algorithm was used to deal with long-range electrostatic interactions and a cut-off value of 12 Å was used to control the van der Waals interactions, respectively. The timestep was set to 2 fs. Finally, the production run has been continued from the NPT ensemble by unrestraining the protein backbone for 50 ns. Temperature and pressure were controlled by the modified Berendsen thermostat (V-rescale) and the Parrinello-Rahman barostat. During the production run, the frames were collected at every 50 ps interval for further data analysis. RMSD, H-bonds, H-bond distance, and H-bond angle of each protein-ligand complex were calculated using the functions *‘gmx rms’, ‘gmx hbond’, ‘gmx distance’* and *‘gmx angle’ built* in Gromacs. The simulation protocol was applied to all protein-ligand complexes.

### MMPBSA binding free energy calculation

The Molecular Mechanics Poison-Boltzmann Surface Area (MMPBSA) is a popular technique to calculate the binding-free energy between the protein and ligand. We have used the g_mmpbsa (Kumari et al., 2014) package to generate the MMPBSA model of the protein-ligand complex, as described in the earlier study (Keretsu, Ghosh & Cho, 2020). After the total MMPBSA binding free energy was calculated, the residue-wise binding energy decomposition calculation was performed.

The binding-free energy from the MMPBSA method can be expressed as –

\begin{eqnarray*}\Delta {G}_{bind}=\Delta {G}_{complex}-\Delta {G}_{protein}-\Delta {G}_{ligand}=\Delta {E}_{MM}+\Delta {G}_{sol}-T\Delta S\nonumber\\\displaystyle \hspace*{100.00015pt}=\Delta {E}_{vdW}+\Delta {E}_{ele}+\Delta {G}_{GB}+\Delta {G}_{SA}-T\Delta S \end{eqnarray*}

In which, the free energy of the protein-ligand complex was expressed by the Δ*G*_*complex*_. The binding energy of the protein and ligand in the solvent was expressed by Δ*G*_*protein*_ and Δ*G*_*ligand*_. The Δ*E*_*MM*_ represents the interaction energy between the protein and ligand in the gas phase condition, which was obtained by calculating the van der Waals energy (Δ*E*_*vdW*_) and electrostatic energy (Δ*E*_*ele*_). The Δ*G*_*sol*_ denote the free energy solvation which was derived by computing the polar solvation Δ*G*_*GB*_ and non-polar solvation Δ*G*_*SA*_ energy. Finally, *T*Δ *S* represents the entropy contribution during the ligand binding to its receptor. Due to the extensive computational process and relatively small impact on the final binding energy calculation, the −*T*Δ*S* term was ignored in this study.

The interaction energy (IE) was calculated by ‘*gmx energy’* function in Gromacs. The IE was estimated by combining the short-range Coulombic interaction (*Coul-SR*) and short-range Lennard Jones (*LJ-SR*) energy between the protein and ligand.

IE can be expressed as

\begin{eqnarray*}IE=Coul-SR+LJ-SR. \end{eqnarray*}

### Dataset building and molecular alignment

In our current study, we have taken 42 novel pyridinyl-benzamide-based compounds which were reported as ROCK1 inhibitors. The inhibitory activity (IC_50_) values, which were in µM units, were converted to log IC_50_ (pIC_50_) values. The converted pIC_50_ values covered the activity range of 3 log units (pIC_50_ = 5.02 to 8.52) for the 3D-QSAR study. The average structure of C08 from the last 1 ns MD trajectory, was taken as a representative compound from the dataset. Based on the structure of the reference molecule, the rest of the compounds were sketched, added hydrogen atoms, and calculated the Gasteiger-Hückel partial atomic charges in SYBYL-X 2.1 (Tripos Inc.) to build the dataset. These compounds were minimized using the tripos force field with a convergence of 0.05 kcal/mol by setting the maximum iteration to 2000 runs. The dataset molecules were further divided into the training set of 32 compounds to build the model and the test set of 9 compounds to evaluate the external predictivity of the 3D-QSAR model. Compound C16 has a nonspecific value and was excluded from the dataset during the 3D-QSAR study.

Molecular alignment of the compounds is a vital step in 3D-QSAR, as it was assumed that, sharing the common scaffold, the compounds would bind to the receptor in a similar orientation (Bang & Cho, 2004). We have used the substructure-based alignment, distill-rigid fit, and database alignment functionality available in SYBYL-X 2.1. After that, the aligned compounds were subjected to the 3D-QSAR study.

### 3D-QSAR study

CoMFA and CoMSIA are two routinely used 3D-QSAR methods to establish the structure–activity relationships of chemical compounds (Gadhe et al., 2011; Pasha et al., 2007). In both methods, the descriptors are calculated in a three-dimensional cubic lattice with 2 Å grid spacing around the molecules. Steric (S) and electrostatic (E) fields were calculated separately for each molecule in CoMFA, by taking *SP*^3^ carbon as a probe with a charge value of +1 and an energy tolerance cutoff of 30 kcal/mol. The Lennard-Jones and Coulomb potential functions were used to calculate the steric and electrostatic fields, respectively. In the CoMSIA model, together with the steric and electrostatic fields, three additional descriptors such as hydrophobic (H), hydrogen bond donor (D), and hydrogen bond acceptor (A) have been calculated. A Gaussian-type function was assigned to all grid points for calculating the similarity indices. The rest of the parameters were kept similar to the CoMFA.

### PLS calculation, CoMFA, and CoMSIA model building and validation

The partial least squares (PLS) method was used to derive the statistically significant CoMFA and CoMSIA models, followed by the cross-validation (*q*^2^) and non-cross validation (*r*^2^) of the training set. In the PLS method, the pIC_50_ values (dependent variables) of the training set were correlated with their respective CoMFA and CoMSIA descriptor fields (independent variables). Leave-one-out (LOO) was used to obtain the cross-validation coefficient, *q*^2^*, the* standard error of prediction (SEP), and the optimal number of components (ONC). Subsequently, a non-cross-validated correlation coefficient, *r*^2^, was generated for statistical indexing of the predictive power of the CoMFA and CoMSIA models along with the Fisher’s statistics (F-value), standard error of estimation (SEE), and field contribution values of each descriptor. The column filtering value was set to 2.0 kcal/mol for both cross and non-cross validation methods. In CoMSIA, different combinations of descriptors (S, E, H, A, and D) were used to obtain the best statistically significant model (San Juan & Cho, 2007).

Sometimes, higher values of *q*^2^ and *r*^2^ may not be sufficient to evaluate the predictive outcomes of CoMFA and CoMSIA. Thus, the external predictive correlation coefficient (*r*^2^_*pred*_) was carried out to assess the predictive power of the models. The predictive correlation coefficient is expressed by the following equation:

\begin{eqnarray*}{r}_{pred}^{2}=(\text{SD-PRESS})/\mathrm{SD} \end{eqnarray*}

SD stands for the sum of the squared deviation between the pIC_50_ values of the test set and the mean activity values of the training set. PRESS is the sum of the squared deviations between the observed and calculated activity values of each test set of compounds.

### Contour map analysis

The CoMFA and CoMSIA result was represented as a StDev*Coeff contour map to visualize the field effect of the chemical descriptors around the most selective compound C08. The green and yellow contours represented the sterically favorable and unfavorable regions, while the blue and red contours represented the favorable substitution for positively or negatively charged chemical groups, respectively, in CoMFA and CoMSIA (Gadhe, Kothandan & Cho, 2012). Likewise, the favorable and unfavorable positions of the hydrophobic, H-bond acceptor and H-bond donor were represented by yellow-white, magenta-red, and purple-cyan by color scheme, respectively.

### New compound design, ADME/Tox, and synthetic accessibility prediction

Based on the CoMFA and CoMSIA contour maps, we have designed forty new compounds and predicted their activity (pIC_50_) by the CoMFA and CoMSIA models. Compounds with higher pIC_50_ values were subjected to docking, MD simulation, and MMPBSA analysis.

The newly designed compound has also proceeded with the ADME/Tox prediction and synthetic accessibility analysis by pkCSM (Pires, Blundell & Ascher, 2015) (http://biosig.unimelb.edu.au/pkcsm) and SwissADMET (Daina, Michielin & Zoete, 2017) (http://www.swissadme.ch) online web server.

## Results

### Molecular docking analysis

**Figure 2:**
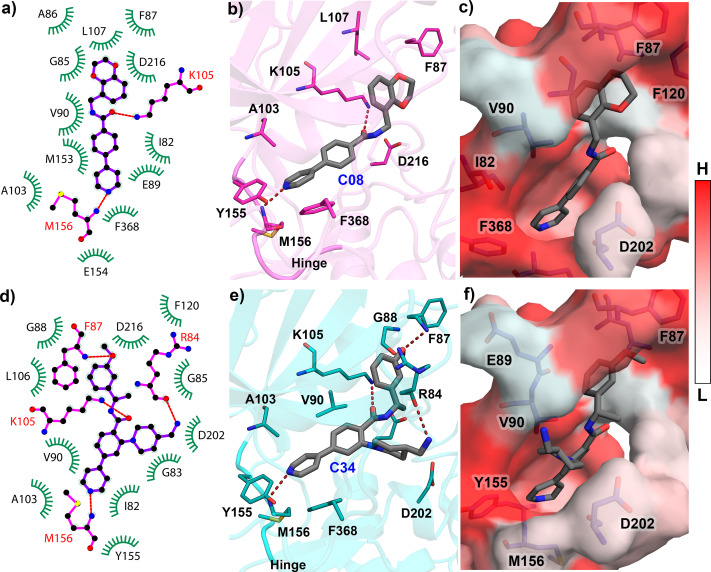
Molecular Docking analysis of compounds C08 and C34 with ROCK1. (A) Docking interaction of C08 in 2D representation. (B) H-bond formation of C08 in the binding pocket. (C) C08 surrounded by hydrophobic residues. (D) Docking interaction of C34 in 2D representation. (E) H-bond formation of C34 in the active site cavity. (F) C34 surrounded by hydrophobic residues. The low (L) to high (H) hydrophobicity was represented by a white to red color scheme. H-bonds have been shown in red dashed lines.

The reliability of the molecular docking was verified by the ligRMSD (Velázquez-Libera et al., 2020) server, as the docking pose was further used in the MD study. The docked structures of ten compounds C03, C08, C11, C13, C19, C22, C25, C29, C34, and C37 were compared by aligning them with the C34 crystal pose (PDB ID: 6E9W). The final docked pose of each compound was selected based on the ECIDALs (Muñoz Gutierrez et al., 2016) norms with an RMSD tolerance of 2 Å. The 1-(4-(pyridin-4-yl)phenyl)ethan-1-one scaffold of the docked compounds and crystal ligand was superimposed at the same position. The RMSD of each compound and their binding energy from the docking study were tabulated in Table-S1. Except for compound C22 (RMSD = 2.89 Å), the other compounds have shown the RMSD value of less than 2 Å from the crystal ligand. The redocking of the C08 showed that it was stabilized by several polar and hydrophobic interactions in the active site. It formed the H-bond interaction with M156, located in the hinge loop, and another H-bond interaction with the catalytic lysine K105. The Gatekeeper residue F368 formed the *π*- *π* interaction with the pyrimidine ring. Other residues, such as I82, F87, A103, L107, Y155, formed the *π*-alkyl and hydrophobic interactions. When docking with the most active compound C34, an increased number of H-bonds was observed. Along with M156 and K105, residues R84 and F87 also participated in the H-bond interaction with C34 through their backbone hydroxyl (-OH) and amine (-NH2) groups. The detailed molecular docking interactions of compounds C08 and C34 have been illustrated in Fig. 2. The 2D molecular docking interactions of the rest of the compounds C03, C11, C13, C19, C22, C25, C29, C37 were shown in Fig. S2. Residues that participated in hydrophobic and H-bond interactions were shown in green and magenta colors, respectively, in Figs. 2A and 2D. The H-bond interactions were shown in red dotted lines in Figs. 2B and 2E. The active site cavity was shown by the hydrophobic surface model based on the Eisenberg scale in Figs. 2C and 2F, where white to red indicated the increase in hydrophobicity. All docked protein-ligand complexes were taken for the MD simulation study.

### MD simulation analysis

**Figure 3:**
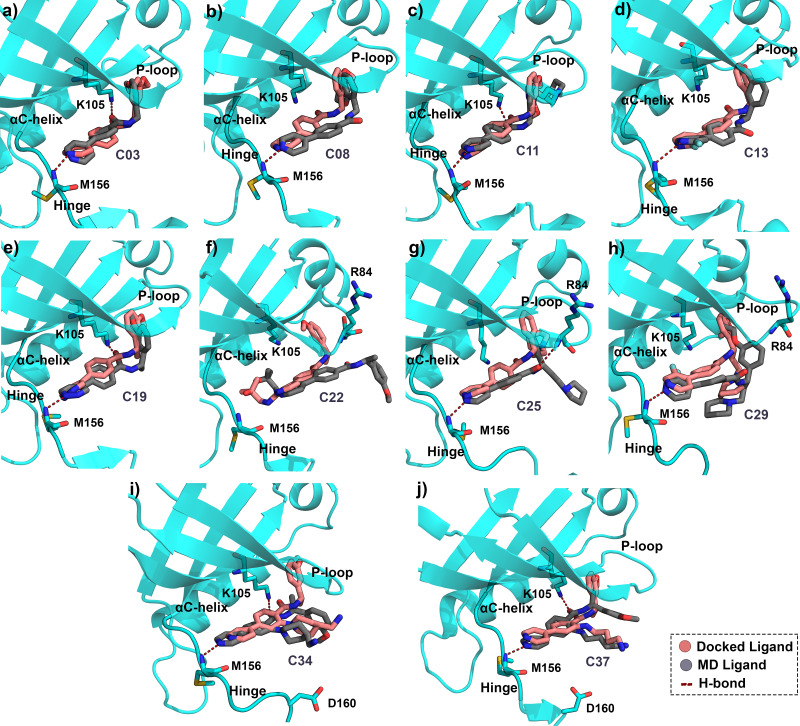
Average MD pose of the ligands inside the ROCK1 active site. Final 1 ns average MD pose of the compound (A) C03, (B) C08, (C) C11, (D) C13, (E) C19, (F) C22, (G) C25, (H) C29, (I) C34, and (J) C37 in dark-grey respectively inside the ROCK1 binding pocket. The corresponding docked pose of each compound was shown in salmon color. The H-bond interactions have been marked by red dashed lines.

Since protein-ligand binding is a dynamic process, and a single docking result would have remained inconclusive. Therefore, we performed MD simulations for all ten protein-ligand complexes for 50 ns. All systems were equilibrated within the initial 10 ns of simulation and thereafter maintained a stable plateau until the end of the run. The RMSD plot of each protein-ligand system was shown in Fig. S3. The fluctuation of RMSDs for the protein was observed within the range of 1.0–4.5 Å, whereas the fluctuations of the ligands were found to be in the range of 0–3.0 Å, respectively. To study the in-depth molecular interaction, we compared the binding pose from the MD trajectory (average structure taken from the final 1 ns) and the docking pose of each compound by superimposing them. The morphed complexes were shown in Fig. 3.

The conformations of compounds C03, C11, C19, C34, and C37 (Figs. 3A, 3C, 3E, 3I, 3J) from the MD simulation displayed close similarity with the corresponding binding pose from the docking study. The H-bond interactions with K105 and M156 remained intact until the final step of the trajectory. In contrast, compounds C08, C13, C25, and C29 Figs. 3B, 3D, 3G, 3H) lost the H-bond interaction between the carbonyl group (-C =0) and K105. However, the H-bond interaction with the backbone nitrogen of M156 remained intact. In addition, compound C25 formed the H-bond interaction with the backbone nitrogen atom (N-atom) of R84 by the carbonyl group. For C22, which is the least active compound in the dataset, the two H-bond interactions with K105 and M156 were found to be broken in the MD simulation analysis.

**Figure 4:**
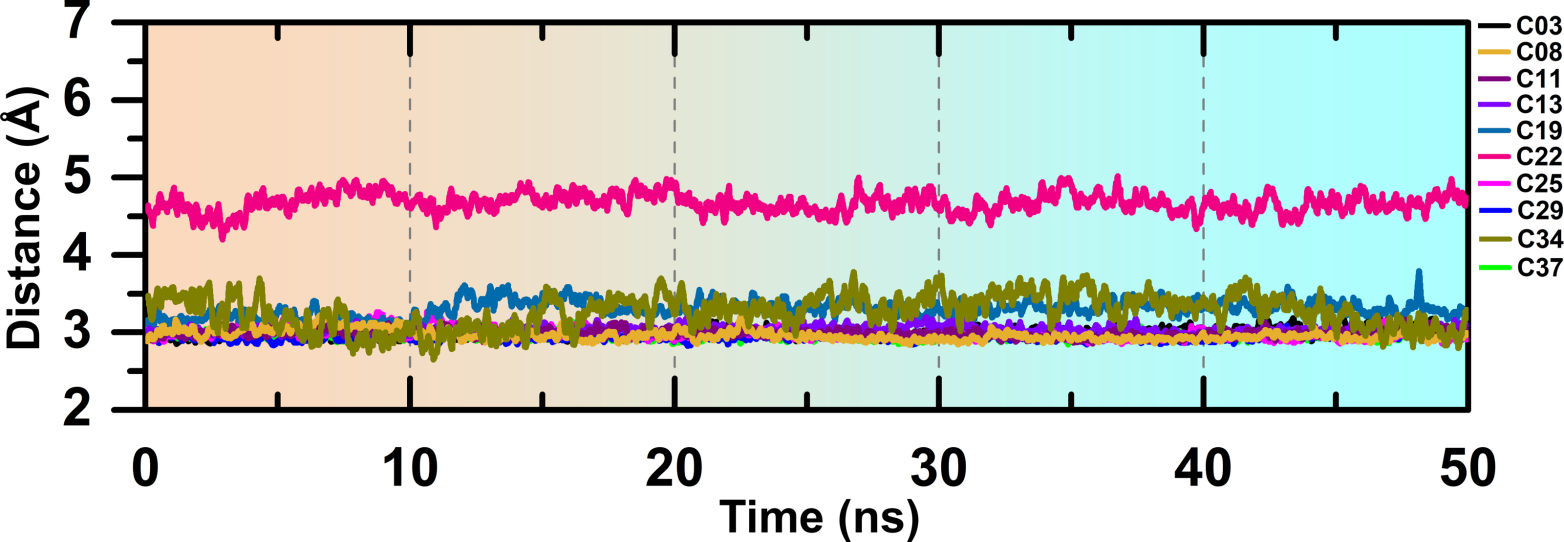
H-bond interaction distance between the M256 and selected compounds during the MD run.

The overlapped structure (Fig. 3F) showed that the distance between the hinge loop and the pyrimidine-2-amine ring of C22 increased and the methoxybenzene moiety was flipped outward from its docked position. However, C22 remained in the active site throughout the production run, mainly by forming polar and hydrophobic interactions with the surrounding residues. Because M156 is the key residue in forming the H-bond interaction, we examined the distance between the N atom of the M156 backbone and the N atom of the pyrimidine ring of the compounds throughout the simulation in Fig. 4. Aside from C22, we found that the H-bond interaction distances from the residue M156 varied between 2–3.3 Å for other compounds. In the case of compound C22, the H-bond interaction distance was increased to ∼4.9 Å. However, we did not see any major changes in the RMSD plot of C22 as observed in the Fig. S3F. One of the key precedents to determine the existence of the H-bond formation is the calculation of the angle between the donor hydrogen and acceptor (D-H-A) atoms, which was expected to be linear or close to 180°. Figure S4 depicted the distribution of the angle formation among the D-H-A atoms for each compound. Furthermore, we have calculated the relative frequency of the total number of H-bond interactions between ROCK1 and selected compounds in Fig. S5. Throughout the production simulation, the frequency of H-bond interactions between ROCK1 and selected compounds was found to be in the range of 0 to 4. C19 and C22 showed a higher frequency of completely broken H-bond interactions (10% and 16%, respectively) compared to other compounds.

### MMPBSA binding energy and IE estimation

**Figure 5:**
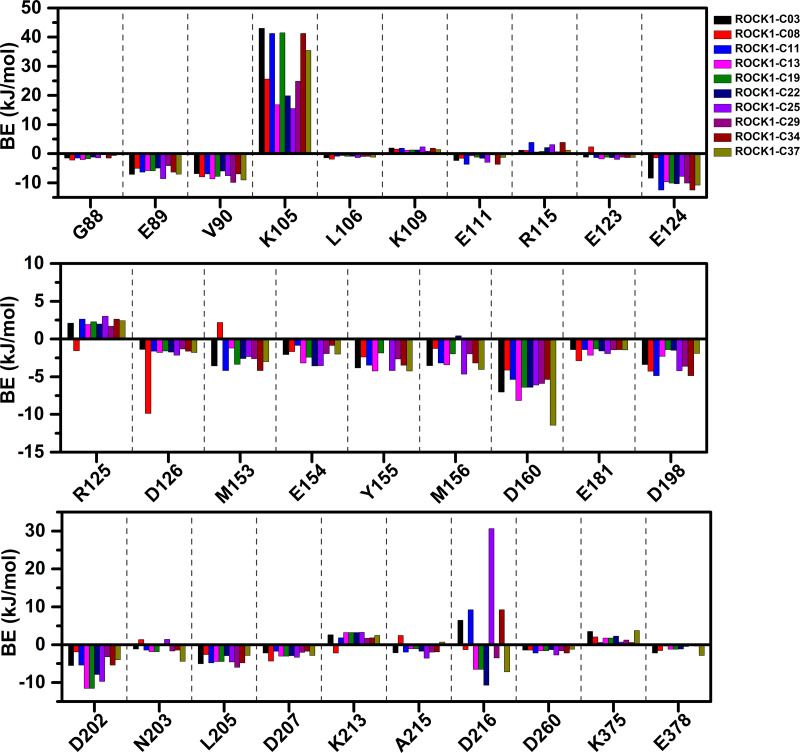
Residue-wise MMPBSA binding energy decomposition in kJ/mol.

We have calculated the MMPBSA binding-free energy and IE from the snapshots extracted from the final 2 ns of the MD trajectory of each system, to estimate the protein-ligand binding affinity. Electrostatic energy, van der Waals energy, and SASA energy terms were the main contributing factors to total BE. Alternatively, the short-range Coulombic (Coul-SR) interaction and the short-range Lennard-Jones (LJ-SR) energy contributed to the total interaction energy. The details of MMPBSA binding free energy and IE were presented in Table 1. The total MMPBSA binding free energies were found to be −107.85 kJ/mol, −109.22 kJ/mol, −114.08 kJ/mol, −102.36 kJ/mol, −76.09 kJ/mol, −95.44 kJ/mol, −123.18 kJ/mol, −108.85 kJ/mol, −130.61 kJ/mol, −106.69 kJ/mol for compounds C03, C08, C11, C13, C19, C22, C25, C29, C34 and C37, respectively. The IE was found to be −253.41 kJ/mol, −247.97 kJ/mol, −279.69 kJ/mol, −194.26 kJ/mol, −215.37 kJ/mol, −210.93 kJ/mol, −284.75 kJ/mol, −226.21 kJ/mol, −284.21 kJ/mol, −233.93 kJ/mol for the compounds C03, C08, C11, C13, C19, C22, C25, C29, C34 and C37, respectively. The most active compound, C34 resulted in the lowest binding-free energy in the MMPBSA model. In addition, with C25, C34 provided the lowest IE values among the other compounds. However, we did not find any correlation between the pIC_50_ and MMPBSA or IE values. The common residues, which contributed to the noticeable amount of net positive and net negative BE energy were shown in Table S2. The graphical comparison of BE decomposition by these residues was shown in Fig. 5. Residues E89, V90, E124, D126, Y155, D160, D202 contributed the lowest BE, while residues K105 and R125 contributed the highest BE to the ligands. However, we observed a large variation in BE decomposition by residue D216. In the formation of the complex with C03, C11, C25, and C34, it provided net positive BE values in the total binding energy. Conversely, it contributed negative BE in compounds C08, C13, C19, C22, C29, and C37, respectively.

**Table 1:** MMPBSA and IE estimation of the compound C03, C08, C11, C13, C19, C22, C25, C29, C34 and C37.

**#Cpd.**	**MMPBSA binding energy terms in kJ/mol**					**Interaction Energy (IE) in kJ/mol** (±SE)
	**Van der Waals energy** (± SD)	**Electrostatic energy** (± SD)	**Polar solvation energy**(± SD)	**SASA energy** (± SD)	**Total Binding energy** (± SD)	
**C03**	−172.3 ± 4.2	−74.6 ± 5.0	158.1 ± 2.0	−19.2 ± 0.2	**−107.8** ± 2.9	**−253.41** ± 1.7
**C08**	−164.2 ± 10.3	−31.2 ± 4.5	103.7 ± 6.4	−17.7 ± 0.3	**−109.2** ± 4.6	**−247.97** ± 2.5
**C11**	−194.7 ± 5.2	−81.4 ± 1.7	186.7 ± 10.8	−23.1 ± 0.3	**−114.0** ± 9.6	**−279.69** ± 3.6
**C13**	−157.0 ± 3.9	−28.7 ± 3.3	102.5 ± 4.5	−19.0 ± 0.2	**−102.3** ± 1.8	**−194.26** ± 2.9
**C19**	−158.8 ± 0.4	−68.2 ± 0.4	169.1 ± 0.6	−18.1 ± 0.1	**−76.0** ± 0.7	**−215.37** ± 5.2
**C22**	−138.2 ± 0.6	−36.9 ± 0.6	96.9 ± 1.0	−17.2 ± 0.1	**−95.4** ± 0.8	**−210.93** ± 1.7
**C25**	−189.8 ± 4.7	−50.2 ± 1.2	138.4 ± 4.8	−21.5 ± 0.9	**−123.1** ± 0.3	**−284.75** ± 2.2
**C29**	−165.5 ± 2.1	−49.2 ± 1.2	125.3 ± 4.9	−19.6 ± 0.1	**−108.8** ± 7.8	**−226.21** ± 4.1
**C34**	−203.3 ± 0.6	−76.6 ± 0.4	172.9 ± 0.7	−23.5 ± 0.1	**−130.6** ± 0.7	**−284.21** ± 4.8
**C37**	−186.8 ± 1.0	−48.9 ± 1.4	148.8 ± 8.6	−19.8 ± 0.2	**−106.6** ± 6.9	**−233.93** ± 3.7

**Notes.**

**#Cpd.** Compounds SD Standard Deviation SE Standard Error

**Table 2:** Structure and activity values of N-ethyl-4-(pyridin-4-yl)benzamide based ROCK1 inhibitor.

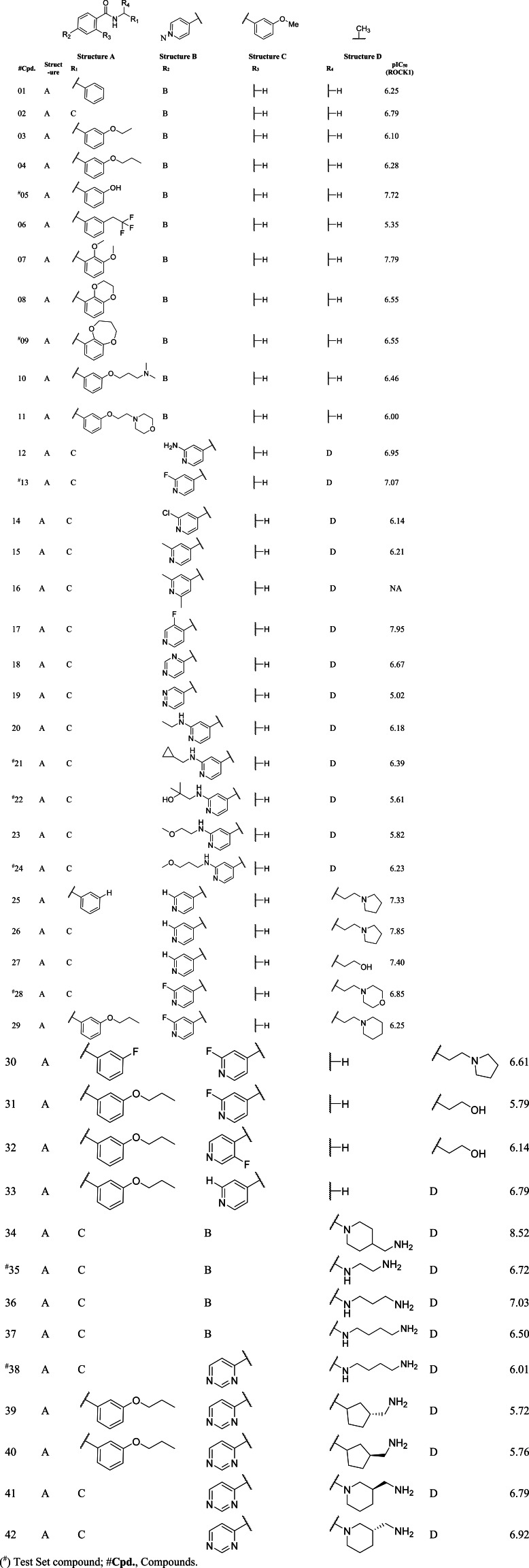

### Molecular alignment and 3D-QSAR studies

The coherence selection of the compounds and the splitting of them into a training set and a test set is an important step toward the development of the 3D-QSAR. The dataset compounds and their corresponding pIC_50_ values were shown in Table 2. We indexed the compounds into high, medium, and low activity ranges and chose the test set compounds so that they can cover different activity values while maintaining structural variations. All training set compounds were aligned to the representative compound C08 by their common substructure N-isopropylbenzamide. The aligned compounds and their common substructure has been illustrated in Fig. S6.

We have produced the CoMFA and CoMSIA models based on the 32 compounds in the training set. For model validation, we have restricted the statistical parameters of *q*^2^ >0.6; *r*^2^ >0.8 and *r*^2^_*pred*_ >0.5. Initially, we applied different charge fields, namely, Gasteiger, Gasteiger-Hückle, Pullman, MMFF94 charges, to obtain the best possible CoMFA model. In the cross-validated model, we obtained the final *q*^2^ = 0.774, an ONC value of 6, and a SEP value of 0.425 in the Gasteiger charge field. In the non-cross-validated model, the *r*^2^ value and the SEE value were found to be 0.965 and 0.167, respectively. We have employed the same charge to build a reliable CoMSIA model with multiple combinations of steric (S), electrostatic (E), hydrophobic (H), and H-bond acceptor (A) and H-bond donor (D) fields.

Only the steric and electrostatic field produced a reasonable CoMSIA model with *q*^2^ = 0.676 and ONC = 6 from cross-validation and *r*^2^ = 0.949 from non-cross-validation, respectively. The *r*^2^_*pred*_ of CoMFA and CoMSIA (SE) was found to be 0.703 and 0.548, which is higher than the set criteria of 0.5. Overall, both CoMFA and CoMSIA exhibited the robustness of the external predictivity. The detailed statistics of the 3D-QSAR statistics were shown in Table 3. The statistics of the different combinations of descriptors in the CoMSIA model have been tabulated in Table S3. The actual pIC_50_ and predicted pIC_50_ from CoMFA and CoMSIA with their residuals were shown in the Table S4.

### CoMFA and CoMSIA contour map and SAR analysis

**Figure 6:**
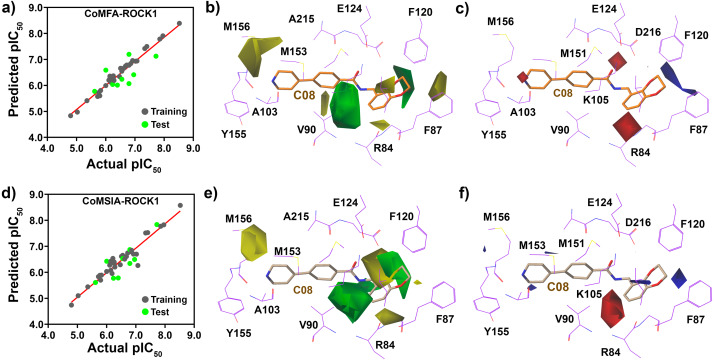
PLS regression plot and Contour map assessment from CoMFA and CoMSIA around the C08. (A) PLS statistical plot from CoMFA, where training sets and test sets are shown by forest-green and light-green color. (B) and (C) are the contour maps, displaying the favorable substitution for the steric and electrostatic chemical groups. Green contour represents the favorable regions for a bulky group, and yellow contour represents the unfavorable regions of that substitution. Blue contour favored the positively charged chemical group, and red contour unfavored that substitution in CoMFA analysis. (D) PLS statistical plot from CoMSIA, where training sets and test sets are shown by forest-green and light-green color. (E) and (F) are the contour maps, displaying the favorable substitution for the steric and electrostatic substitution. Green contour signifies the favorable regions for a bulky group, and yellow contour signifies the sterically unfavorable regions for that substitution. Blue contour favored the positively charged electrostatic substitution, whereas, red contour unfavored that substitution in CoMSIA analysis.

From the 3D-QSAR study, we have plotted the correlation plots between the actual pIC50 and the predicted pIC_50_ in Fig. 6. The compounds in the training and test sets have been labeled dark and light green, respectively. The contour maps generated by CoMFA and CoMSIA around the compound C08 are shown in Figs. 6B, 6C, 6E and 6F. From the CoMFA model, we obtained the steric and electrostatic field contributions of 63% and 37%, respectively, and from the CoMSIA model, we found the steric and electrostatic field contribution of 57.2% and 42.8%, respectively. A green contour map indicates a favorable substitution for the bulky steric groups, whereas a yellow contour indicates that the steric substitution would be unfavorable. Similarly, a blue and red contours represent favorable and unfavorable substitutions for positively charged electron-donating groups, respectively, to increase the inhibitory activity of the molecules. The green contour was observed at position R_4_ (Figs. 6B and 6E) near F120, which indicated that the steric groups could be favorable at that position. Compounds C25 and C26 with a propylpyrrolidine moiety at the R_4_ position exhibited higher activity values than Compounds C01-C04 and C08-C11 with smaller groups present at their R_4_ position. A green contour at the R_3_ position near residue V90 also indicated a favorable substitution for the steric groups. The yellow contours were observed near the M156 position in R_2_ and near the R84 residue in R_1_ position in the CoMFA model, which represented the unfavorable position for steric substitution. The contour maps suggested that a steric group would be unfavorable for R_2_ substitution. This could be the reason why compounds C34, C36, and C25-27 had higher activities than C20-24. Figures 6C and 6F show a blue contour covering the dioxane ring at the R_1_ position, and two red contours near K105 and R84, indicating that the electron-donating and electron-accepting group replacement may increase the effectiveness of the inhibitors.

### Designing of new compounds

**Figure 7:**
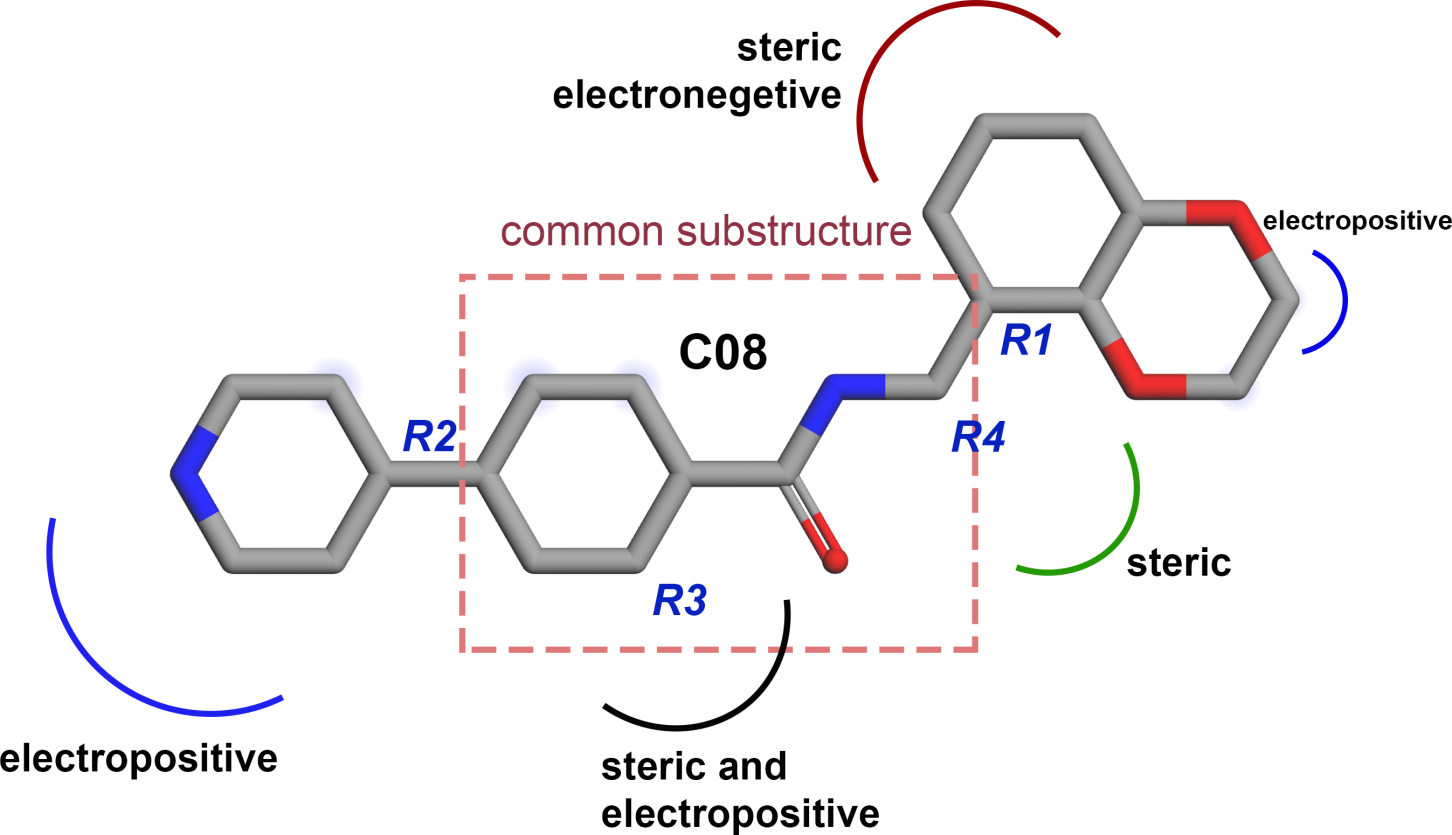
Diagram of the three-dimensional structure–activity relationship of the representative compound C08.

**Figure 8:**
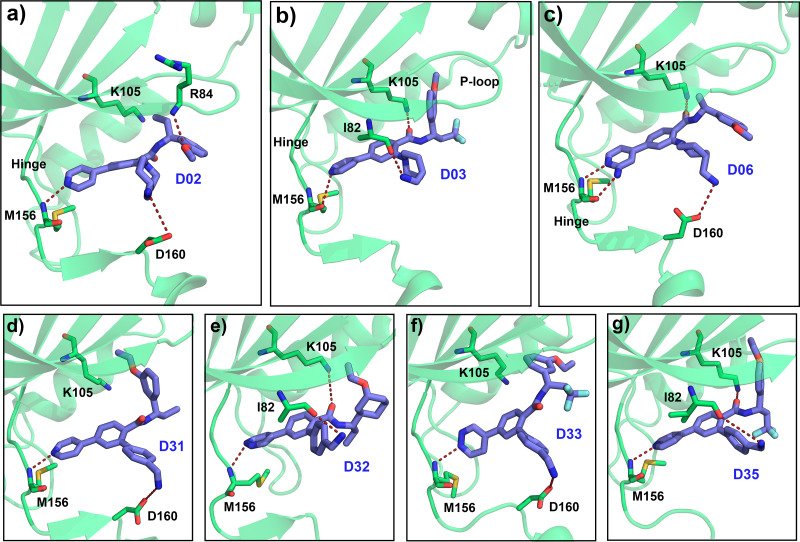
Average MD pose of the designed compound in the ATP pocket. The last 1 ns average MD pose of the designed compound (A) D02, (B) D03, (C) D06, (D) D31, (E) D32, (F) D33, and (G) D35 respectively. The H-bond interaction was shown by red dashed lines.

Based on the CoMFA and CoMSIA contour map analysis, we have acquired an ideal SAR scheme design a more potent ROCK1 inhibitor drawn in Fig. 7, based on compound 08. A steric and an electropositive group would be preferred for the R_2_ and R_4_ positions. A steric-electropositive group at the R_3_ position could increase the activity of existing compounds. As the CoMFA scheme showed higher statistical significance (*q*^2^ and *q*^2^) and predictive power (*q*^2^_*pred*_), the designed compounds were evaluated by the CoMFA model. Based on the scheme in Fig. 7, we have designed forty new compounds and predicted their activity values in the CoMFA model, Table S5. Of these, seven designed compounds, D02, D03, D06, D31, D32, D33, D35, have exhibited higher pIC_50_ values than the most active compound C34, as shown in Table 4. We have utilized the pkCSM and SwissADME server to analyze the ADME/Tox and SA scores. For comparison, we have also taken the compounds C08, C34, and C37 from the dataset. The detailed analysis has been tabulated in Table S6. Except for compound D06, none of the designed compounds showed AMES toxicity. The SA score predicts the synthetic accessibility of small molecules. A SA score of 1 denotes the ease of synthesis and an SA score of 10 expresses the difficulty of synthesizing the chemical compound. The SA score of the designed compound was predicted to be less than 5, which stated that the compounds would have a low to moderate difficulty in the synthesis routes. We have also examined the docking (Table S7) and MD studies on these seven newly designed compounds to estimate their binding interaction. The RMSD graph was shown in Fig. S7. The MMPBSA and the interaction energy were shown in Table 5. The residue-wise binding energy decomposition was shown in Table S9. The catalytic lysine K105 has exhibited a higher positive binding energy decomposition among the selected residues. Finally, we have taken the last 1 ns average structure of the protein and designed ligand complexes to study the key interactions at the residue level. The designed compounds tend to form a higher number of H-bond interactions with the surrounding residues compared to the dataset compounds. The in-depth interactions in the active site pocket were illustrated in Fig. 8.

**Table 3:** Statistics of the selected CoMFA and CoMSIA models.

**Parameters**	**CoMFA**	**CoMSIA (SE)**
***q*** ^2^	0.774	0.676
**ONC**	6	6
**SEP**	0.425	0.509
***r*** ^2^	0.965	0.949
**SEE**	0.167	0.201
**F-value**	114.795	78.237
***r*** ^**2**^_**pred**_	0.703	0.548
**S (%)**	63.0	57.2
**E (%)**	37.0	42.8

**Notes.**

*q*^2^ squared cross-validated correlation coefficient ONC optimal number of components SEP standard error of prediction *r*^2^ squared correlation coefficient SEE standard error of estimation F-value F-test value ${r}_{pred}^{2}$ predictive ***r***^2^ S steric E electrostatic

**Table 4:** Newly designed compounds with higher predicted pIC_50_ values.

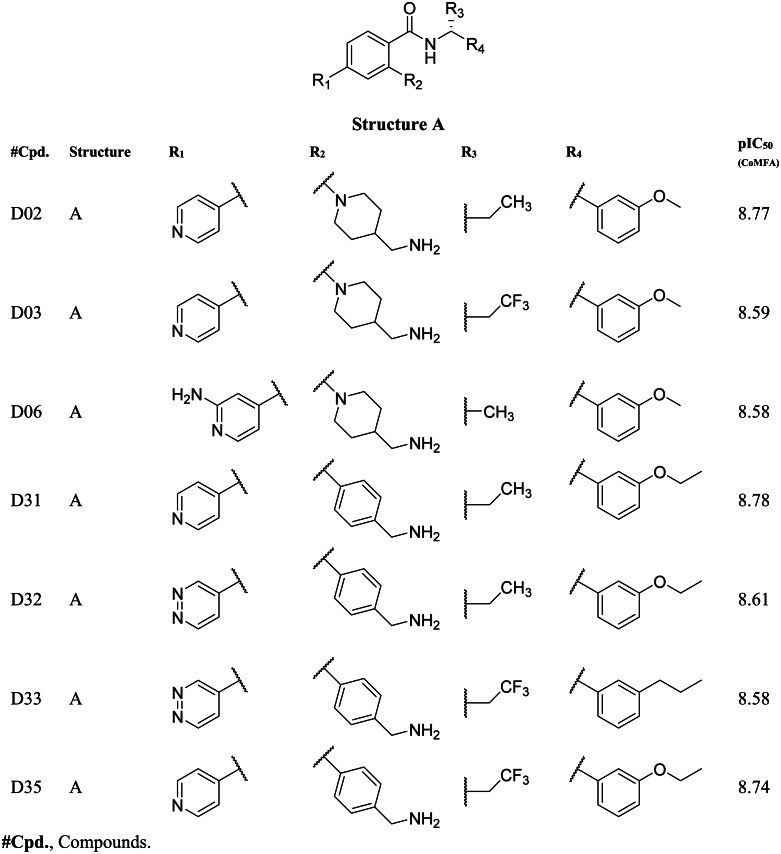

**Table 5:** MMPBSA and IE estimation of the compound D02, D03, D06, D31, D32, D33, and D35.

**#Cpd.**	**MMPBSA binding energy terms in kJ/mol**					**Interaction Energy (IE) in kJ/mol** (±SE)
	**Van der Waals energy** (±SD)	**Electrostatic energy** (±SD)	**Polar solvation energy** (±SD)	**SASA energy** (±SD)	**Total Binding energy** (±SD)	
**D02**	−203.8 ± 6.1	−37.7 ± 3.3	126.6 ± 4.3	−22.2 ± 0.5	**−137.0** ± 2.6	**−245.0** ± 3.4
**D03**	−224.5 ± 2.5	−72.1 ± 1.6	187.0 ± 4.7	−24.9 ± 0.4	**−134.7** ± 8.1	**−280.1** ± 2.7
**D06**	−191.7 ± 4.4	−97.9 ± 2.4	202.8 ± 4.9	−22.7 ± 0.3	**−109.6** ± 5.6	**−223.4** ± 5.9
**D31**	−169.1 ± 0.5	−28.5 ± 0.4	78.7 ± 0.8	−20.2 ± 0.1	**−139.2** ± 0.7	**−204.9** ± 3.9
**D32**	−192.8 ± 4.8	−68.9 ± 2.0	169.6 ± 12.1	−23.0 ± 0.5	**−115.3** ± 5.6	**−227.4** ± 1.4
**D33**	−191.4 ± 2.4	−31.4 ± 1.3	104.0 ± 1.2	−21.3 ± 0.1	**−140.1** ± 0.2	**−214.9** ± 2.4
**D35**	−189.5 ± 2.0	−57.2 ± 3.4	134.2 ± 4.3	−22.7 ± 0.2	**−135.0** ± 5.8	**−248.0** ± 1.1

**Notes.**

**#Cpd.** Compounds SD Standard Deviation SE Standard Error

## Discussion

**Figure 9:**
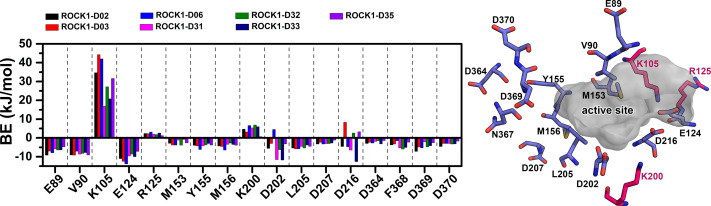
Comparison of the residue-wise MMPBSA binding energy contribution to the newly designed compounds. For the assessment, the residues were selected within the 3.5 Å distance from the ligand. Residues with net positive BE decomposition are shown in hot-pink color, residues with net negative BE decomposition are shown in the slate color. The active site was represented by a grey surface scheme.

Together with the docking study, the CoMFA and CoMSIA contour maps provided a valuable insight into the rational modifications of existing compounds. The data were shown in Tables S1 and S7 from ligRMSD, which has provided the validity of our docking protocol for both the existing compounds and designed compounds. One of the key H-bond interactions was between the M156 residue and the N atom of the pyrimidine ring, which played a crucial role in anchoring the ligands. The surrounding residues were found to be M153, A215, Y155, A103, V90, F120, and F87, which formed the hydrophobic surface. In CoMFA study, a yellow contour was observed near the hinge loop, which suggested that the compound having a steric group might not be favorable. A small blue contour was observed near the backbone of Y155, suggesting that a small electron donor would be advantageous at that site. From the docking and MD study, we have observed that C22, which has a 2-methyl-1- (methylamino) propan-2-ol group with the pyrimidine moiety, prevents the formation of H bonds with M156 and resulted in the loss of the H-bond interaction. This may affect the inhibitory activity of compound C22. For the same reason, having a steric group on the pyrimidine ring, C21, C23, and C24 might have decreased inhibitory activity. Another key H-bond interaction was detected between the K105 and the -C =O groups of the docked compounds. Due to the presence of a rotatable bond (torsion) in the molecules, this H-bond formation might occasionally get interrupted. At the R_3_ position, a large green contour suggested that a bulky steric group will be beneficial. However, our docking and MD studies suggested that substitution for the larger groups could be limited. Similarly, a larger substitution of the steric group at the R_4_ position might result in a steric clash with the surrounding residues. Therefore, we have chosen the major modification strategies at the positions R_3_ and R_4_ when designing the new compounds. The introduction of chemical group substitution at the positions R_3_ and R_4_ increased the probability of H-bond formation with either D160 or I82, which led to the higher binding affinity of the newly designed compounds to ROCK1. The RMSD graphs of the newly designed compounds indicated stable protein-ligand complexes. We have calculated the angular distributions and distances among the D-H-A atoms of M156 and the newly designed compounds in Figs. S8 and S9, respectively. Finally, the average H-bond angle values and distances of the dataset compounds and designed compounds were summarized in Table S8. The distance between the H-bond donor and acceptor atoms was found to be less than 5 Å, and the angle values were found to be within the range of 120°–180°. This configuration suggests a reasonable geometry for a stable H-bond interaction. In the MMPBSA energy evaluation, the vdW energy and the electrostatic energy added the key energy values in total BE. Nonethless, negative SASA scores also favored total binding energy. In Fig. 9 below, we have drawn a comparative plot of the decomposition of the MMPBSA binding energy to the designed compounds. Residues with net negative binding energy contribution and net positive energy contribution were shown in slate and magenta colors. Residues K105, R125, and K200 have been found to contribute a positive energy value to the total binding energy of the designed compounds. Residues E89, V90, E124, D202, L205, and F368 contributed to negative binding energy decomposition.

Prior to the commencement of the experimental procedure, the prediction of the pharmacokinetic properties and bioavailability of the designed compounds is necessary. The prediction of ADME/Tox showed the high gastrointestinal absorption of the designed compounds, which indicated that the compound would have good bioavailability. The inhibitory effects of the designed compounds on different types of Cytochrome P450 were marked by Yes/No representation and possibly excreted through biotransformation. In general, the ADME/Tox analysis predicted that the designed compound would be a safer and more active ROCK1 inhibitor.

## Conclusions

The discovery of a potent ROCK1 inhibitor is a promising strategy to achieve the therapeutic goal against cardiovascular disease and carcinomas. In our present study, we have performed the molecular modeling study of 41 pyridinyl-benzamide-based ROCK1 inhibitors. Molecular docking, Molecular Dynamics, MMPBSA, and IE calculations provide critical information about the molecular interactions and binding affinity between protein-inhibitor complexes. We have established a reasonable correlation between the actual and predictive activity of the compounds from the CoMFA and CoMSIA models with external predictive capability. The contour maps from the CoMFA and CoMSIA described the structure–activity relationship of the compounds. Models also suggest that it could predict the activity of the newly designed compound with a similar scaffold. Based on the SAR study, we have designed several new compounds, and seven of them expressed higher pIC_50_ values compared to the most active compound C34. Estimation of ADME/Tox and SA scores suggested that the designed compounds would have more potent inhibitory activity against ROCK1 while having the desirable pharmacokinetics property and bioavailability. Additionally, we have validated our results with the selected designed compounds by MD simulation, MMPBSA, and IE models. The outcome of our study might be useful to the future development of the ROCK1 inhibitor.

##  Supplemental Information

10.7717/peerj.11951/supp-1 Supplemental Information 1 Supplementary Figures and Tables

10.7717/peerj.11951/supp-2 Supplemental Information 2 Protein-ligand docked complex, input/output files, ligand topology-parameter files, forcefield, and running scripts

## Additional Information and Declarations

### Data Availability

The following information was supplied regarding data availability: The raw data is available in the Supplementary Files and at Zenodo: Suparna Ghosh, Seketoulie Keretsu, & Seung Joo Cho. (2021). Designing of the ROCK1 inhibitors. http://doi.org/10.5281/zenodo.5077971.
