# Supplementary material for: Designing of the N-ethyl-4-(pyridin-4-yl)benzamide based potent ROCK1 inhibitors using docking, molecular dynamics, and 3D-QSAR
Source: PeerJ. 2021 Aug 9;9:e11951. doi: 10.7717/peerj.11951 (PMC8359802; doi:10.7717/peerj.11951)
Supplement: Supplemental Information 1 [file peerj-09-11951-s001.docx]

**Supplementary Data: Designing of the N-ethyl-4-(pyridin-4-yl)benzamide based potent ROCK1 inhibitor using Docking, Molecular Dynamics, and 3D-QSAR**

Suparna Ghosh ^1^, Seketoulie Keretsu ^1^, Seung Joo Cho ^1,2, 🖂^

^1^Department of Biomedical Sciences, College of Medicine, Chosun University, Gwangju 501-759, Republic of Korea; [s.ghosh@chosun.kr (S.G)](mailto:s.ghosh@chosun.kr%20(S.G)); keretsu@chosun.kr (S.K); [chosj@chosun.ac.kr](mailto:chosj@chosun.ac.kr) (S.J.C)

^2^Department of Cellular and Molecular Medicine, College of Medicine, Chosun University, Gwangju 501-759, Republic of Korea

^🖂^Corresponding Author:

Seung Joo Cho

Gwangju 501-759, Republic of Korea

Email address: chosj@chosun.ac.kr;

Tel.: +82-62-230-7482 (office) or +82-11-479-1010 (cell phone)


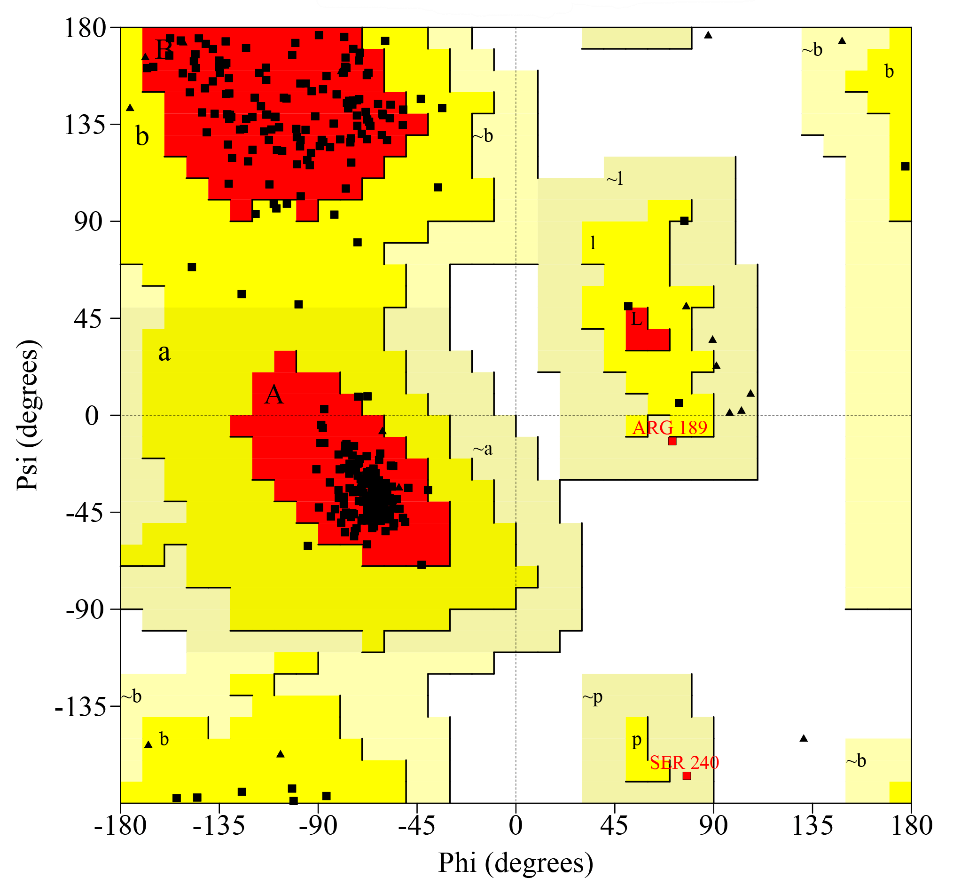


**Supplementary Figure-S1. Ramachandran Plot analysis of ROCK1 by PROCHECK**

Modeled loops are in the well accepted region.


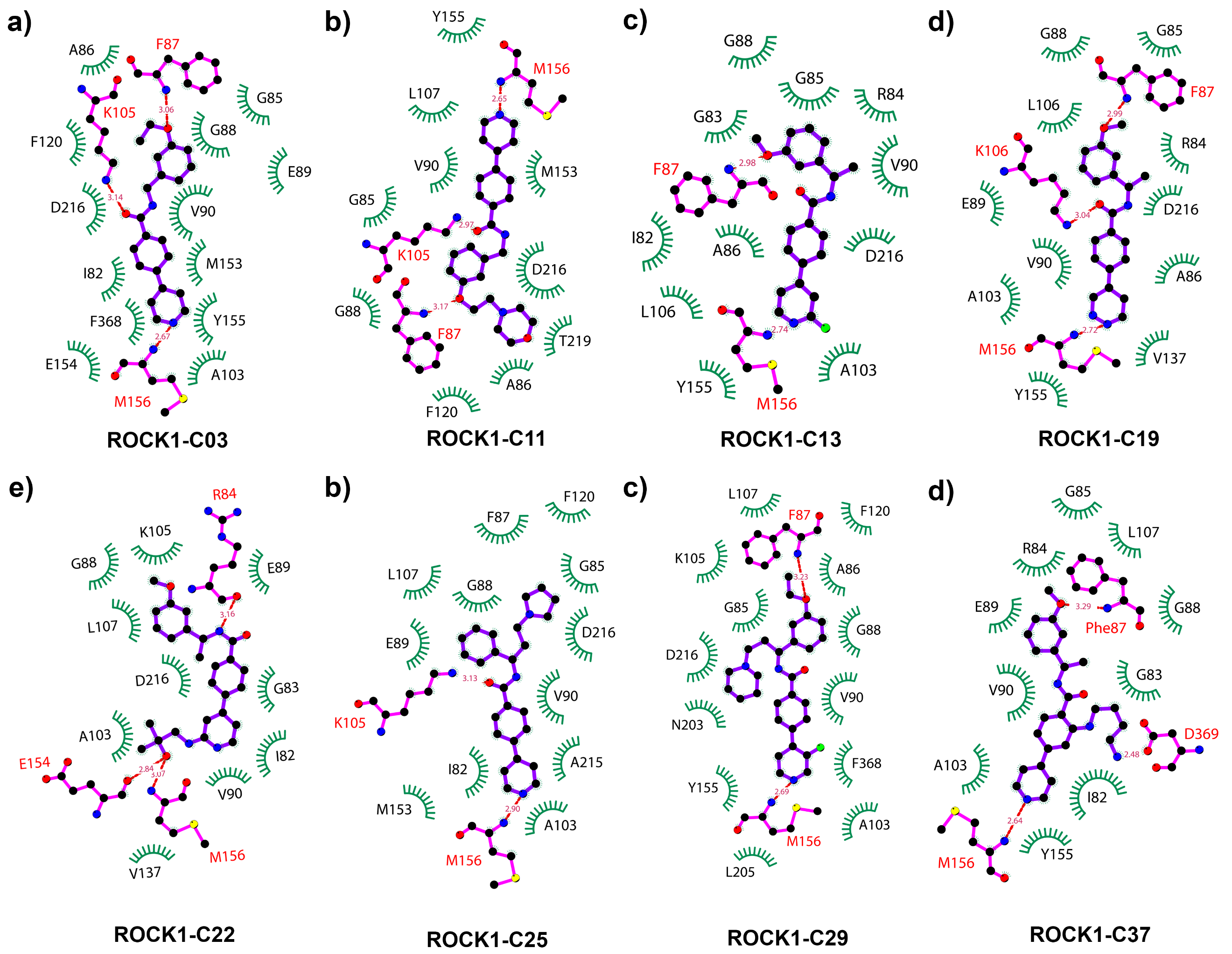


**Supplementary Figure-S2. Molecular Docking analysis of compound C03, C11, C13, C19, C22, C25, C29 and C37**

Hydrophobic residues were shown in green color, compounds were shown in violet color and H-bonds have been shown in red-dashed lines.

**Supplementary Table-S1. ligRMSD and Docking energy evaluations of the compounds**

| Molecules | Match Type | RMSD (Å) | % ref match | % molecule match | ΔG (kcal.mol^-1^) |
| --- | --- | --- | --- | --- | --- |
| C03 | strict | 0.53 | 84.6 | 88.0 | -10.14 |
| C08 | ’’ | 1.84 | 100.0 | 100.0 | -9.20 |
| C11 | ’’ | 0.67 | 84.6 | 70.9 | -11.57 |
| C13 | ’’ | 0.88 | 84.6 | 70.9 | -9.67 |
| C19 | ’’ | 0.60 | 65.3 | 68.0 | -9.70 |
| C22 | ’’ | 2.89 | 84.6 | 70.9 | -9.21 |
| C25 | ’’ | 0.60 | 84.6 | 75.8 | -9.95 |
| C29 | ’’ | 1.22 | 84.6 | 64.7 | -10.51 |
| C34 | ’’ | 0.54 | 84.6 | 66.6 | -12.49 |
| C37 | ’’ | 1.24 | 84.6 | 70.9 | -13.40 |

RMSDs were calculated from the crystal pose of C08. ΔG was calculated from AutoDock4.2.

**
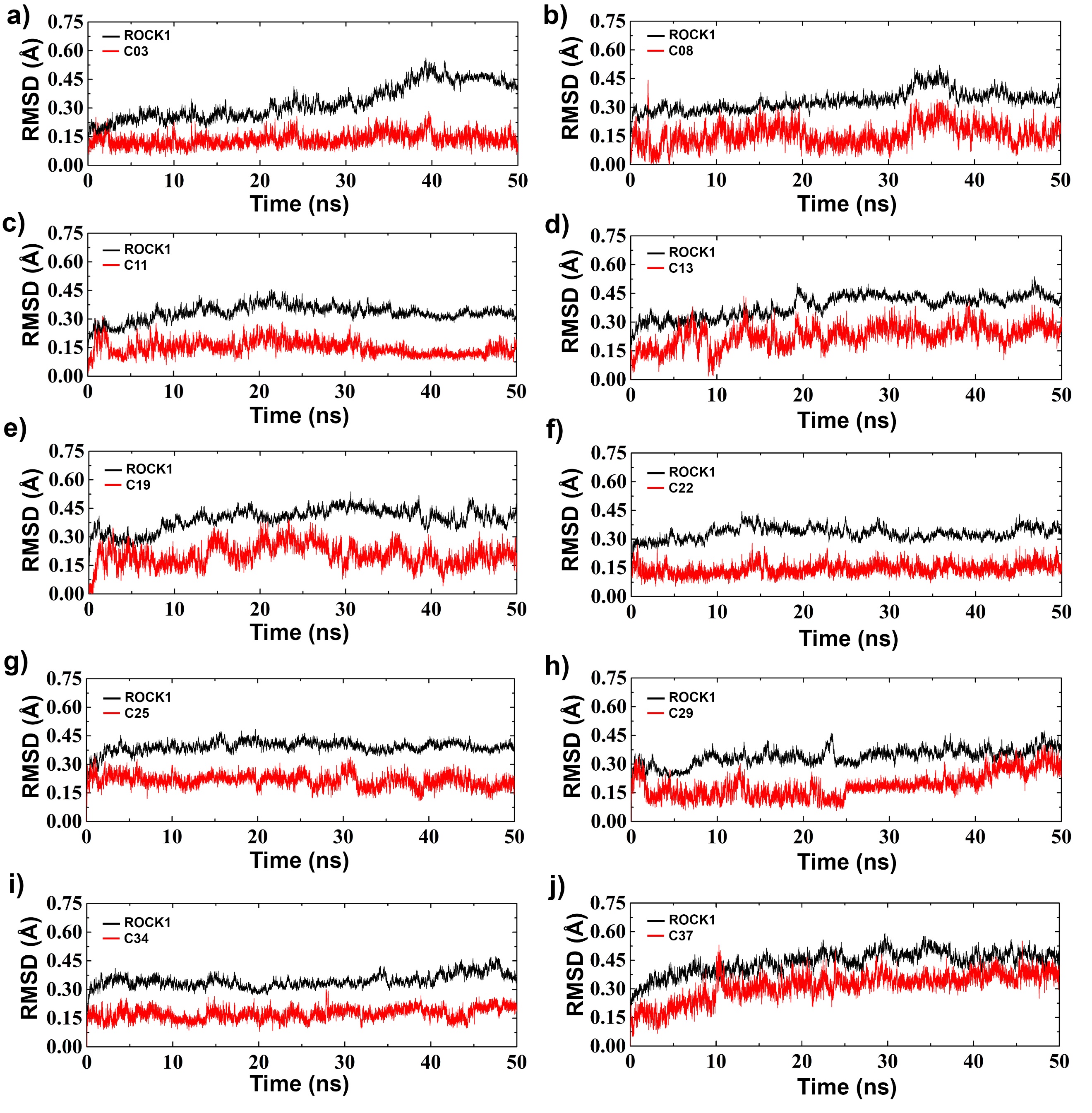
**

**Supplementary Figure-S3. RMSD analysis of compound C03, C08, C11, C13, C19, C22, C25, C29, C34 and C37**

**
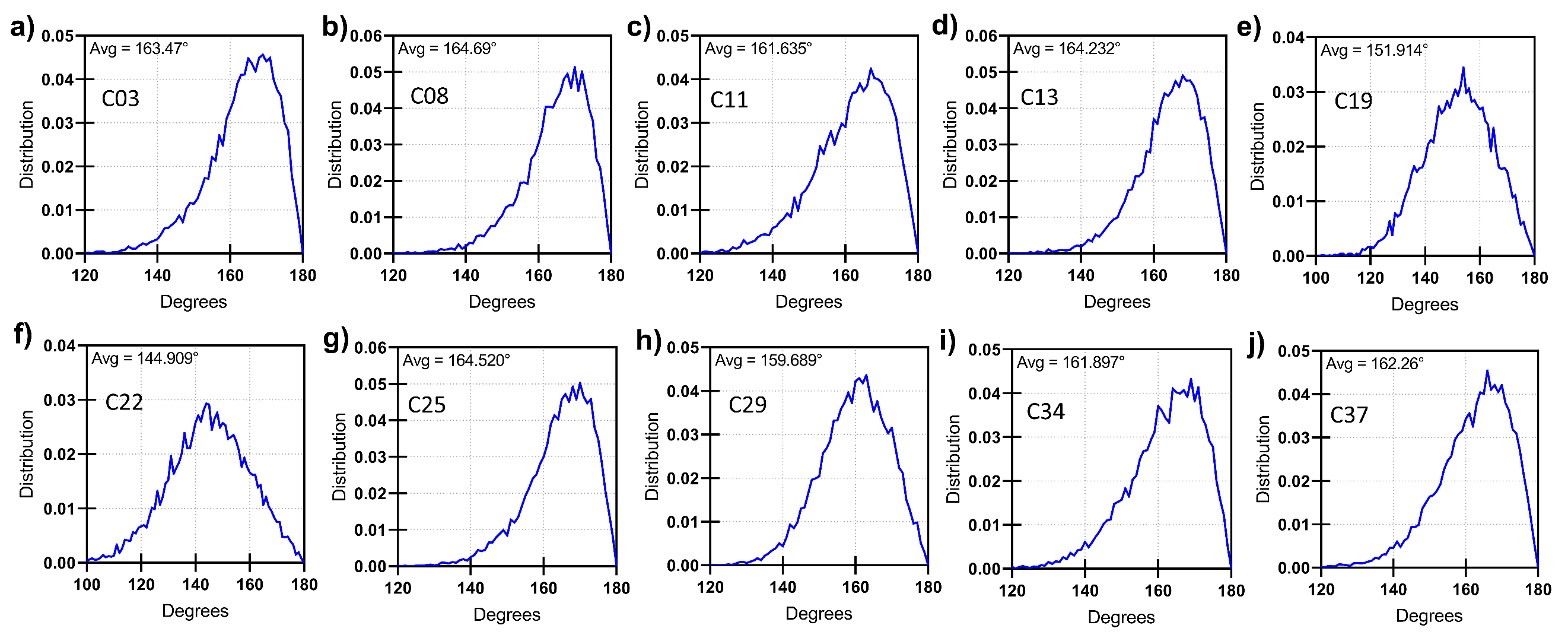
Supplementary Figure-S4. Angular distribution of the H-bond interaction between the residue M156 and compounds a) C03, b) C08, c) C11, d) C13, e) C19, f) C22, g) C25, h) C29, i) C34 and j) C37 during the MD simulation**

**
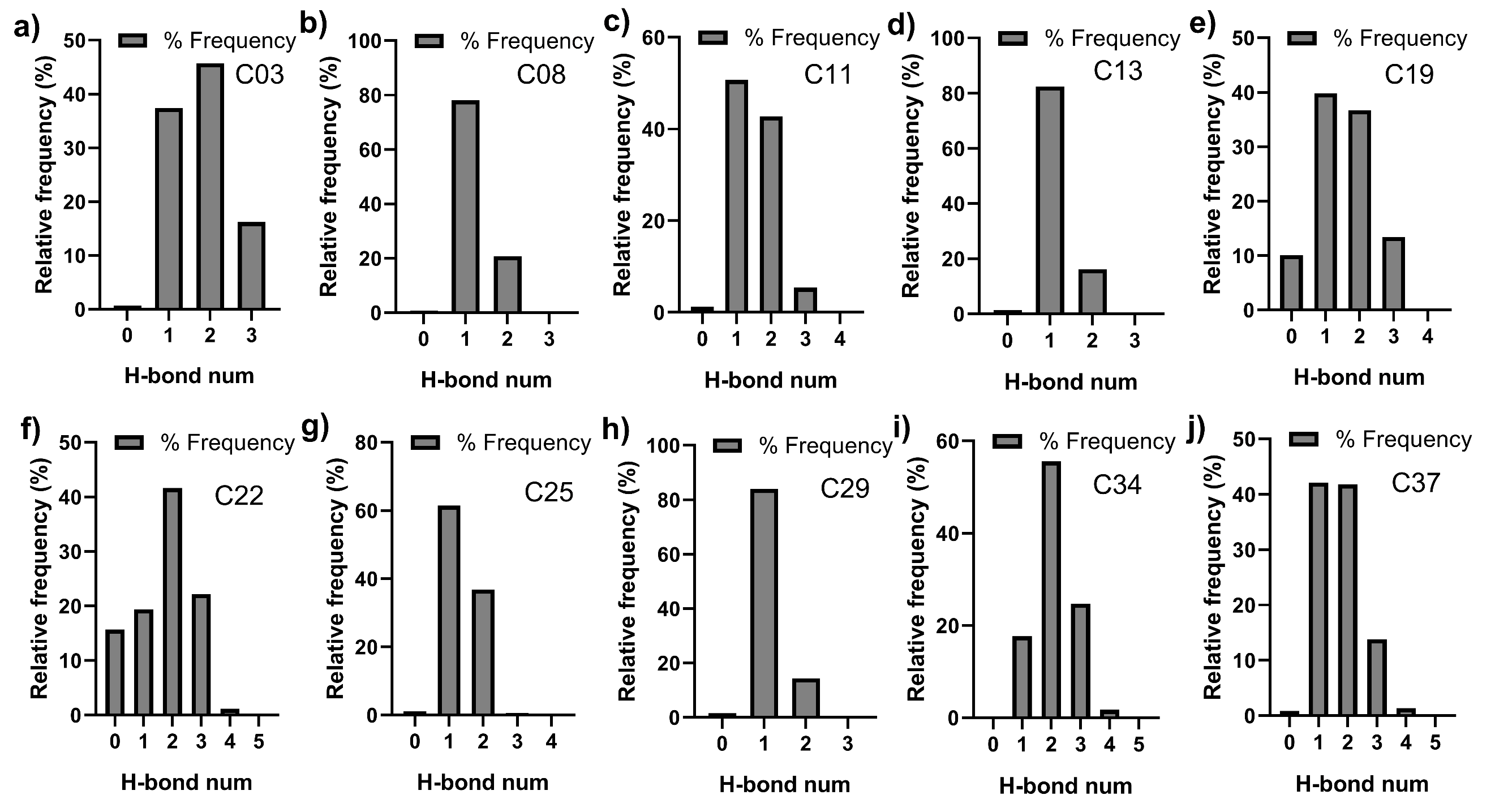
**

**Supplementary Figure-S5. Relative frequency (%) of H-bond formation between ROCK1 and compounds a) C03, b) C08, c) C11, d) C13, e) C19, f) C22, g) C25, h) C29, i) C34 and j) C37 during the production simulation**

For C19 and C22, the relative frequency of broken H-bond interactions was appeared to be higher than other compounds at the ROCK1 active site (10% and 16%, respectively).

**Supplementary Table-S2. MMPBSA binding energy decomposition from the residues in kJ/mol**

| **Residue** | **C03** | **C08** | **C11** | **C13** | **C19** | **C22** | **C25** | **C29** | **C34** | **C37** |
| --- | --- | --- | --- | --- | --- | --- | --- | --- | --- | --- |
| **G88** | -1.48 | -2.17 | -1.48 | -2.04 | -1.73 | -1.22 | -1.42 | 0.43 | -1.48 | -0.66 |
| **E89** | -7.11 | -5.01 | -6.37 | -5.81 | -5.90 | -4.98 | -8.53 | -4.15 | -6.37 | -7.05 |
| **V90** | -6.90 | -7.92 | -6.93 | -8.61 | -7.76 | -5.96 | -7.58 | -9.88 | -6.93 | -9.04 |
| **K105** | 43.04 | 25.59 | 41.27 | 16.83 | 41.51 | 19.84 | 15.47 | 24.85 | 41.28 | 35.46 |
| **L106** | -1.46 | -1.92 | -0.90 | -0.64 | -0.90 | -0.86 | -1.33 | -0.97 | -0.90 | -1.22 |
| **K109** | 1.97 | 1.59 | 1.90 | 1.16 | 1.25 | 1.28 | 2.34 | 0.83 | 1.90 | 1.38 |
| **E111** | -2.33 | -1.58 | -3.62 | -0.78 | -1.21 | -1.60 | -2.93 | -0.47 | -3.62 | -1.25 |
| **R115** | 1.20 | 1.07 | 3.87 | 0.58 | 0.80 | 2.11 | 3.06 | 0.67 | 3.87 | 1.15 |
| **E123** | -1.17 | 2.29 | -1.37 | -1.80 | -1.20 | -1.36 | -1.98 | -1.18 | -1.37 | -1.25 |
| **E124** | -8.40 | -1.41 | -12.46 | -9.61 | -10.07 | -10.32 | -7.81 | -10.09 | -12.45 | -10.80 |
| **R125** | 2.10 | -1.57 | 2.64 | 1.91 | 2.27 | 1.99 | 3.01 | 1.69 | 2.64 | 2.41 |
| **D126** | -1.38 | -9.88 | -1.63 | -1.79 | -1.59 | -1.72 | -2.15 | -1.31 | -1.63 | -1.79 |
| **M153** | -3.55 | 2.19 | -4.17 | -1.19 | -3.36 | -2.59 | -2.32 | -2.61 | -4.17 | -2.99 |
| **E154** | -2.06 | -1.68 | -0.84 | -3.18 | -2.41 | -3.57 | -3.52 | -1.93 | -0.84 | -1.99 |
| **Y155** | -3.84 | -2.38 | -3.47 | -4.23 | -1.84 | -0.11 | -4.19 | -2.64 | -3.47 | -4.25 |
| **M156** | -3.52 | -1.27 | -3.17 | -3.40 | -1.96 | 0.41 | -4.65 | -1.96 | -3.17 | -4.04 |
| **D160** | -7.03 | -4.11 | -5.35 | -8.17 | -6.39 | -6.39 | -6.09 | -5.88 | -5.35 | -11.42 |
| **E181** | -1.41 | -2.88 | -1.41 | -2.14 | -1.31 | -1.60 | -1.94 | -1.44 | -1.41 | -1.44 |
| **D198** | -3.37 | -4.27 | -4.87 | -2.29 | -1.42 | -1.49 | -4.22 | -3.62 | -4.86 | -1.90 |
| **K200** | 4.02 | -1.28 | 5.04 | 5.29 | 3.02 | 0.90 | 6.75 | 4.94 | 5.04 | 2.57 |
| **D202** | -5.52 | -1.90 | -5.38 | -11.52 | -5.31 | -7.86 | -9.71 | -3.23 | -5.38 | -3.96 |
| **N203** | -1.16 | 1.33 | -1.46 | -1.87 | -1.04 | -0.16 | 1.40 | -1.67 | -1.45 | -4.36 |
| **L205** | -5.01 | -2.62 | -4.80 | -4.47 | -3.80 | -2.88 | -4.55 | -5.95 | -4.80 | -2.87 |
| **D207** | -2.22 | -4.34 | -1.75 | -3.03 | -2.21 | -2.91 | -3.34 | -2.00 | -1.75 | -2.87 |
| **K213** | 2.65 | -2.20 | 1.81 | 3.24 | 2.25 | 3.19 | 3.26 | 1.65 | 1.81 | 2.46 |
| **A215** | -2.15 | 2.42 | -1.94 | -1.09 | 1.17 | -1.74 | -3.56 | -1.96 | -1.93 | 0.71 |
| **D216** | 6.43 | -1.34 | 9.23 | -6.52 | 7.84 | -10.72 | 30.65 | -3.48 | 9.24 | -7.13 |
| **D260** | -1.44 | -1.42 | -2.20 | -1.62 | -1.22 | -1.33 | -2.71 | -1.61 | -2.20 | -1.22 |
| **K375** | 3.49 | 2.04 | 0.54 | 1.72 | 1.02 | 2.27 | 0.67 | 1.23 | 0.54 | 3.72 |
| **E378** | -2.17 | -1.53 | -0.38 | -1.17 | -0.95 | -1.15 | -0.51 | -0.15 | -0.38 | -2.86 |


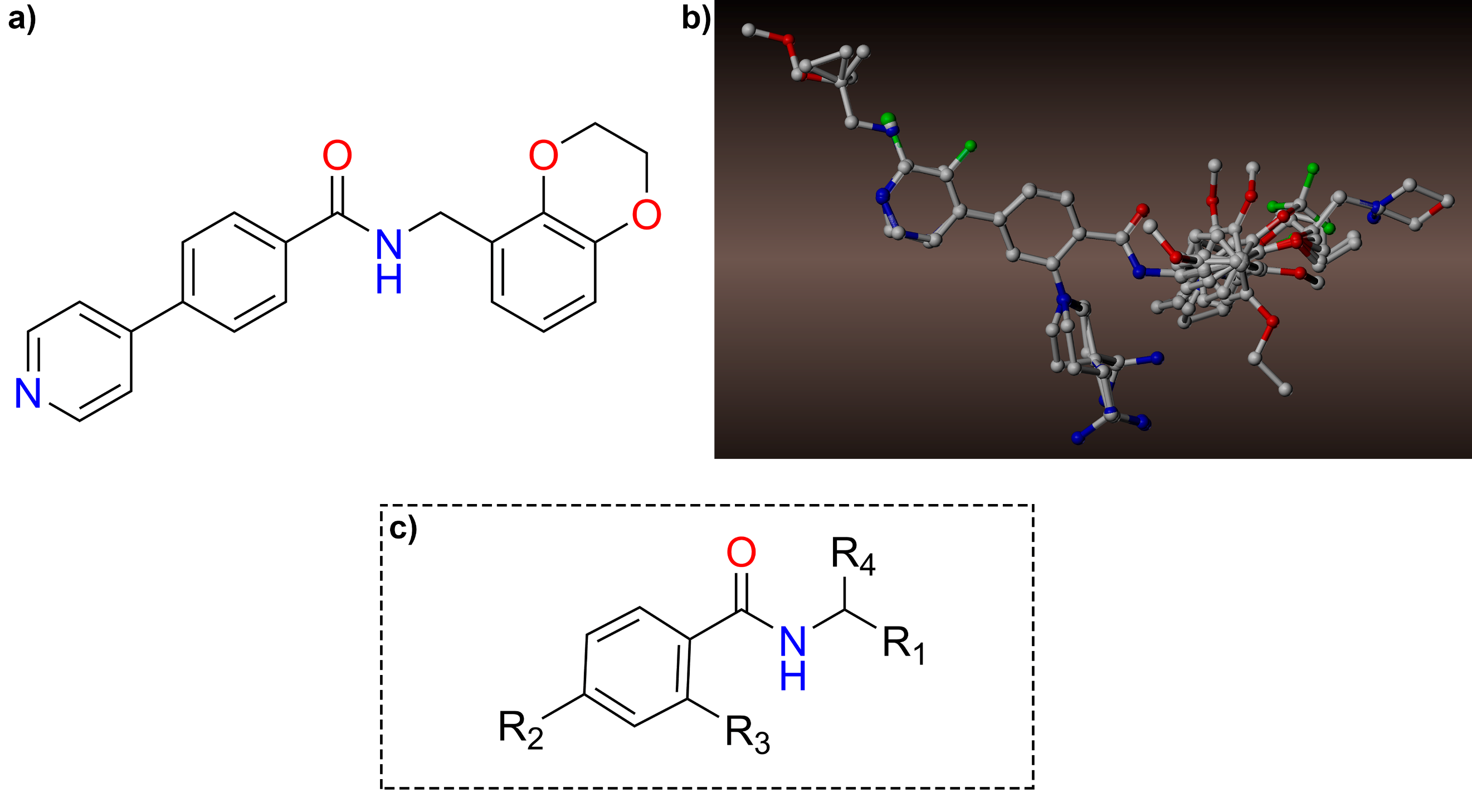


**Supplementary Figure-S6. Molecular alignment of the dataset compounds**

a) Compound C08 was taken as a representative molecule of the dataset. **b)** Molecular alignment of the dataset compounds and **c)** the common substructure of the molecules in the dataset.

**Supplementary Table-S3. Detail statistical value of the CoMSIA models**

| **CoMSIA** | ***q^2^*** | **ONC** | **SEP** | ***r^2^*** | **SEE** | **F-value** | **Field Contribution** | | | | |
| --- | --- | --- | --- | --- | --- | --- | --- | --- | --- | --- | --- |
|  |  |  |  |  |  |  | **S** | **E** | **H** | **A** | **D** |
| S | 0.585 | 6 | 0.576 | 0.915 | 0.260 | 45.060 | 1 | - | - | - | - |
| E | 0.411 | 6 | 0.686 | 0.888 | 0.299 | 32.930 | - | 1 | - | - | - |
| H | 0.101 | 4 | 0.815 | 0.948 | 0.203 | 76.214 | - | - | 1 | - | - |
| A | -0.275 | 2 | 0.937 | 0.396 | 0.694 | 2.733 | - | - | - | 1 | - |
| D | -0.213 | 1 | 1.285 | 0.399 | 0.693 | 2.763 | - | - | - | - | 1 |
| **SE** | **0.676** | **6** | **0.509** | **0.949** | **0.201** | **78.237** | **0.428** | **0.572** | **-** | **-** | **-** |
| EH | 0.385 | 6 | 0.701 | 0.953 | 0.193 | 84.764 | - | 0.611 | 0.389 | - | - |
| EA | 0.359 | 6 | 0.716 | 0.924 | 0.247 | 50.476 | - | 0.740 | - | 0.260 | **-** |
| ED | 0.339 | 6 | 0.727 | 0.959 | 0.181 | 97.310 | - | 0.748 | - | - | 0.252 |
| SH | 0.483 | 6 | 0.663 | 0.953 | 0.194 | 83.840 | 0.510 | - | 0.490 | - | - |
| SA | 0.430 | 6 | 0.674 | 0.890 | 0.297 | 33.625 | 0.673 | - | - | 0.327 | - |
| SD | 0.343 | 6 | 0.724 | 0.907 | 0.272 | 40.712 | 0.633 | - | - | - | 0.367 |
| HA | -0.106 | 6 | 0.940 | 0.895 | 0.290 | 35.498 | - | - | 0.648 | 0.352 | - |
| HD | -0.184 | 6 | 0.972 | 0.899 | 0.284 | 36.981 | - | - | 0.680 | - | 0.320 |
| AD | -0.283 | 1 | 0.924 | 0.501 | 0.631 | 4.186 | - | - | - | 0.528 | 0.472 |
| SHE | 0.546 | 6 | 0.602 | 0.967 | 0.163 | 120.774 | 0.310 | 0.412 | 0.278 | - | - |
| SEA | 0.615 | 6 | 0.555 | 0.956 | 0.187 | 91.106 | 0.335 | 0.451 | - | 0.194 | - |
| SED | 0.530 | 6 | 0.612 | 0.972 | 0.149 | 146.521 | 0.294 | 0.482 | - | - | 0.224 |
| EHA | 0.353 | 6 | 0.719 | 0.960 | 0.180 | 98.934 | - | 0.503 | 0.300 | 0.197 | - |
| EHD | 0.347 | 6 | 0.722 | 0.974 | 0.144 | 157.34 | - | 0.529 | 0.276 | - | 0.196 |
| SHA | 0.366 | 6 | 0.712 | 0.942 | 0.215 | 67.584 | 0.399 | - | 0.368 | 0.233 | - |
| SHD | 0.363 | 6 | 0.713 | 0.944 | 0.212 | 69.973 | 0.404 | - | 0.372 | - | 0.224 |
| EAD | 0.347 | 6 | 0.722 | 0.974 | 0.144 | 157.338 | - | 0.529 | 0.276 | - | 0.196 |
| HAD | -0.313 | 1 | 0.935 | 0.885 | 0.302 | 32.213 | - | - | 0.485 | 0.267 | 0.248 |
| SEHD | 0.496 | 6 | 0.634 | 0.980 | 0.125 | 207.507 | 0.239 | 0.368 | 0.211 | - | 0.181 |
| SEHA | 0.521 | 6 | 0.618 | 0.972 | 0.150 | 144.463 | 0.262 | 0.345 | 0.228 | 0.165 | - |
| SEAD | 0.491 | 6 | 0.638 | 0.966 | 0.157 | 130.695 | 0.274 | 0.403 | - | 0.147 | 0.177 |
| EHAD | 0.236 | 6 | 0.781 | 0.971 | 0.152 | 139.643 | - | 0.455 | 0.237 | 0.149 | 0.158 |
| SHAD | 0.191 | 6 | 0.804 | 0.940 | 0.220 | 64.785 | 0.340 | - | 0.296 | 0.187 | 0.176 |
| SEHAD | 0.447 | 6 | 0.664 | 0.979 | 0.130 | 192.191 | 0.220 | 0.320 | 0.184 | 0.131 | 0.145 |

(Green Highlightes: Best CoMSIA (SE) models; S: Steric; E: Electrostatic; H: Hydrophobic; A: H-bond acceptor; D: H-bond donor)

| **#Cpd** | **CoMFA** | | | **CoMSIA(SE)** | | |
| --- | --- | --- | --- | --- | --- | --- |
|  | **Actual pIC_50_** | **Predicted pIC_50_** | **Residual** | **Actual pIC_50_** | **Predicted pIC_50_** | **Residual** |
| 01 | 6.25 | 6.27 | -0.01 | 6.25 | 6.42 | -0.16 |
| 02 | 6.79 | 6.77 | 0.01 | 6.79 | 6.75 | 0.04 |
| 03 | 6.10 | 6.20 | -0.09 | 6.10 | 6.40 | -0.29 |
| 04 | 6.28 | 6.19 | 0.09 | 6.28 | 6.26 | 0.02 |
| 05 | 7.72 | 7.12 | 0.59 | 7.72 | 7.67 | 0.04 |
| 06 | 5.35 | 5.41 | -0.05 | 5.35 | 5.53 | -0.18 |
| 07 | 4.79 | 4.83 | -0.03 | 4.79 | 4.75 | 0.03 |
| 08 | 6.55 | 6.23 | 0.31 | 6.55 | 6.56 | -0.00 |
| 09 | 6.55 | 6.54 | 0.00 | 6.55 | 6.46 | 0.08 |
| 10 | 6.46 | 6.34 | 0.12 | 6.46 | 6.27 | 0.18 |
| 11 | 6.00 | 5.99 | 0.00 | 6.00 | 6.01 | -0.01 |
| 12 | 6.95 | 6.41 | 0.54 | 6.95 | 6.57 | 0.38 |
| 13 | 7.07 | 6.94 | 0.13 | 7.07 | 6.31 | 0.75 |
| 14 | 6.14 | 6.35 | -0.21 | 6.14 | 6.47 | -0.32 |
| 15 | 6.21 | 6.31 | -0.10 | 6.21 | 6.59 | -0.38 |
| 17 | 7.95 | 7.90 | 0.04 | 7.95 | 7.78 | 0.17 |
| 18 | 6.67 | 6.74 | -0.06 | 6.67 | 6.64 | 0.03 |
| 19 | 5.02 | 4.97 | 0.05 | 5.02 | 5.08 | -0.05 |
| 20 | 6.18 | 6.11 | 0.07 | 6.18 | 6.11 | 0.07 |
| 21 | 6.39 | 6.01 | 0.38 | 6.39 | 5.88 | 0.50 |
| 22 | 5.61 | 5.77 | -0.15 | 5.61 | 5.34 | 0.27 |
| 23 | 5.82 | 5.80 | 0.01 | 5.82 | 5.63 | 0.19 |
| 24 | 6.23 | 6.03 | 0.20 | 6.23 | 5.72 | 0.50 |
| 25 | 7.33 | 7.41 | -0.08 | 7.33 | 7.48 | -0.14 |
| 26 | 7.85 | 7.78 | 0.07 | 7.85 | 7.71 | 0.14 |
| 27 | 7.40 | 7.49 | -0.08 | 7.40 | 7.58 | -0.17 |
| 28 | 6.85 | 6.84 | 0.01 | 6.85 | 6.71 | 0.14 |
| 29 | 6.25 | 6.36 | -0.10 | 6.25 | 6.48 | -0.22 |
| 30 | 6.61 | 6.66 | -0.04 | 6.61 | 6.40 | 0.21 |
| 31 | 5.79 | 5.60 | 0.19 | 5.79 | 5.68 | 0.11 |
| 32 | 6.14 | 6.26 | -0.12 | 6.14 | 6.11 | 0.02 |
| 33 | 6.79 | 6.06 | 0.72 | 6.79 | 6.51 | 0.28 |
| 34 | 8.52 | 8.39 | 0.13 | 8.52 | 8.55 | -0.03 |
| 35 | 6.72 | 6.78 | -0.06 | 6.72 | 6.93 | -0.20 |
| 36 | 7.03 | 6.93 | 0.09 | 7.03 | 6.87 | 0.14 |
| 37 | 6.50 | 6.65 | -0.15 | 6.50 | 6.56 | -0.05 |
| 38 | 6.00 | 6.58 | -0.58 | 6.00 | 6.42 | -0.42 |
| 39 | 5.72 | 5.57 | 0.14 | 5.72 | 5.81 | -0.08 |
| 40 | 5.76 | 5.73 | 0.03 | 5.76 | 5.78 | -0.01 |
| 41 | 6.79 | 7.19 | -0.39 | 6.79 | 6.89 | -0.09 |
| 42 | 6.92 | 6.95 | -0.03 | 6.92 | 6.73 | 0.18 |

**Supplementary Table-S4. Actual pIC_50_ vs predicted pIC_50_ values with their residuals of selected CoMFA and CoMSIA (SE) models**

**Supplementary Table-S5. Newly designed compounds and their predicted pIC_50_ values**

|   **Structure A** | | | | | | |
| --- | --- | --- | --- | --- | --- | --- |
| **#Cpd.** | **Structure** | **R_1_** | **R_2_** | **R_3_** | **R_4_** | **pIC_50 (CoMFA)_** |
| D01 | A |  |  |  |  | 8.34 |
| *D02 | A |  |  |  |  | 8.77 |
| *D03 | A |  |  |  |  | 8.59 |
| D04 | A |  |  |  |  | 7.92 |
| D05 | A |  |  |  |  | 8.48 |
| *D06 | A |  |  |  |  | 8.58 |
| D07 | A |  |  |  |  | 8.34 |
| D08 | A |  |  |  |  | 8.36 |
| D09 | A |  |  |  |  | 8.19 |
| D10 | A |  |  |  |  | 7.78 |
| D11 | A |  |  |  |  | 7.78 |
| D12 | A |  |  |  |  | 8.33 |
| D13 | A |  |  |  |  | 8.17 |
| D14 | A |  |  |  |  | 7.79 |
| D15 | A |  |  |  |  | 7.36 |
| D16 | A |  |  |  |  | 7.36 |
| D17 | A |  |  |  |  | 7.75 |
| D18 | A |  |  |  |  | 7.15 |
| D19 | A |  |  |  |  | 7.33 |
| D20 | A |  |  |  |  | 7.96 |
| D21 | A |  |  |  |  | 7.79 |
| D22 | A |  |  |  |  | 8.17 |
| D23 | A |  |  |  |  | 8.24 |
| D24 | A |  |  |  |  | 7.40 |
| D25 | A |  |  |  |  | 7.46 |
| D26 | A |  |  |  |  | 7.78 |
| D27 | A |  |  |  |  | 7.33 |
| D28 | A |  |  |  |  | 7.24 |
| D29 | A |  |  |  |  | 5.02 |
| D30 | A |  |  |  |  | 8.42 |
| *D31 | A |  |  |  |  | 8.78 |
| *D32 | A |  |  |  |  | 8.61 |
| *D33 | A |  |  |  |  | 8.58 |
| D34 | A |  |  |  |  | 8.39 |
| *D35 | A |  |  |  |  | 8.74 |
| D36 | A |  |  |  |  | 6.01 |
| D37 | A |  |  |  |  | 7.18 |
| D38 | A |  |  |  |  | 7.63 |
| D39 | A |  |  |  |  | 7.68 |
| D40 | A |  |  |  |  | 7.64 |

(*) Designed Compound with higher predictive pIC_50_. **Cpd.:** Compounds; D: Designed

**Supplementary Table-S6. ADME/Tox and Synthetic accessibility predictions**

| **Compounds** | **Absorption** | **Distribution** | **Metabolism** | | | | | | | | **Excretion** | **Toxicity** | **Synthetic Accessibility** |
| --- | --- | --- | --- | --- | --- | --- | --- | --- | --- | --- | --- | --- | --- |
|  | **Intestinal absorption (human)** | **VDss (human)** | **GI absorption** | **P-gp substrate** | **CYP** | | | | | | **Total Clearance** | **AMES toxicity** |  |
|  | **Numeric (% Absorbed)** | **Numeric (log L/kg)** |  |  | **2D6** | **3A4** | **1A2** | **2C19** | **2C9** | **3A4** | **Numeric (log ml/min/kg)** | **Categorical** |  |
| **C08** | 94.08 | 1.28 | High | Yes | No | Yes | No | No | No | Yes | 0.688 | No | 2.67 |
| **C34** | 90.15 | 1.41 | High | Yes | No | Yes | No | Yes | No | Yes | 0.882 | No | 3.90 |
| **C37** | 93.92 | 1.31 | High | Yes | No | Yes | No | No | No | Yes | 0.698 | No | 3.49 |
| **D02** | 91.23 | 1.22 | High | Yes | No | Yes | No | No | No | Yes | 0.355 | No | 4.05 |
| **D03** | 84.63 | 1.43 | High | Yes | No | Yes | No | Yes | No | Yes | 0.632 | No | 4.14 |
| **D06** | 93.19 | -0.16 | High | Yes | No | Yes | No | Yes | No | Yes | 0.729 | Yes | 4.06 |
| **D31** | 90.85 | -0.38 | High | Yes | No | Yes | No | Yes | Yes | Yes | 0.367 | No | 3.64 |
| **D32** | 93.53 | -0.37 | High | Yes | No | Yes | No | Yes | No | Yes | 0.710 | No | 3.89 |
| **D33** | 90.85 | -0.38 | Low | Yes | No | Yes | No | Yes | Yes | Yes | 0.367 | No | 3.97 |
| **D35** | 90.50 | -0.17 | Low | Yes | No | Yes | No | Yes | No | Yes | 0.386 | No | 3.75 |

Absorption (human intestinal absorption), Distribution (VDss), Metabolism (GI absorption, P-gp substrate, and CYP), Excretion (Total clearance), and Toxicity (AMES toxicity) have been predicted using the pKCSM server, whereas, and Synthetic Accessibility has been evaluated using SwisADMET.

**Supplementary Table-S7. ligRMSD and Docking energy evaluations of the compounds**

| Molecules | Match Type | RMSD (Å) | % ref match | % molecule match | ΔG (kcal.mol^-1^) |
| --- | --- | --- | --- | --- | --- |
| D02 | strict | 1.03 | 84.6 | 64.7 | -10.38 |
| D03 | ’’ | 1.99 | 84.6 | 59.4 | -8.83 |
| D06 | ’’ | 0.42 | 84.6 | 64.7 | -12.01 |
| D31 | ’’ | 2.37 | 84.6 | 62.8 | -9.58 |
| D32 | ’’ | 2.22 | 84.6 | 57.8 | -9.90 |
| D33 | ’’ | 2.35 | 84.6 | 57.8 | -14.23 |
| D35 | ’’ | 2.21 | 84.6 | 57.8 | -13.54 |

RMSDs were calculated from the crystal pose of C08. ΔG was calculated from AutoDock4.2.


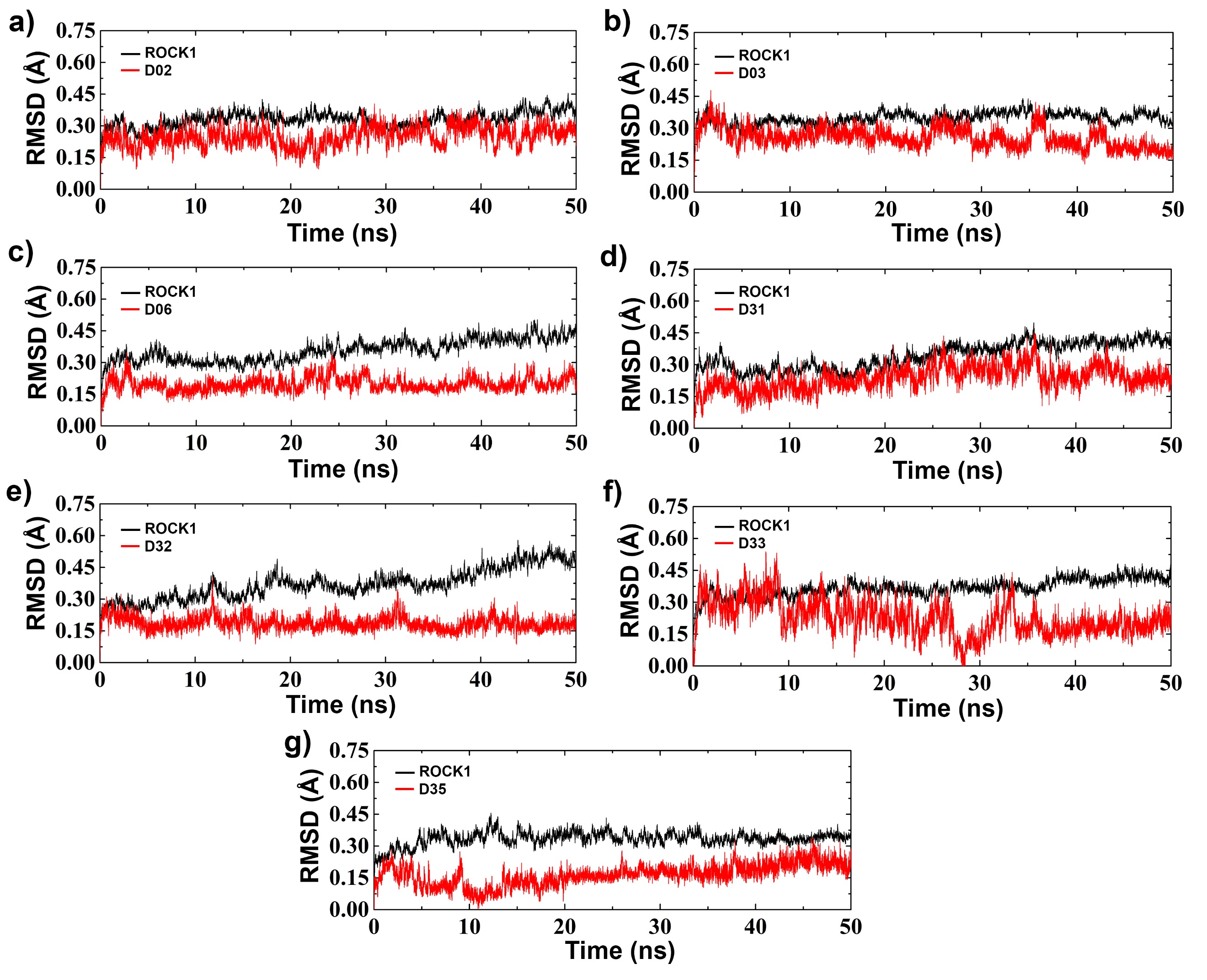


**Supplementary Figure-S7. RMSD analysis of the designed compound D02, D03, D06, D31, D32, D33 and D35**

**
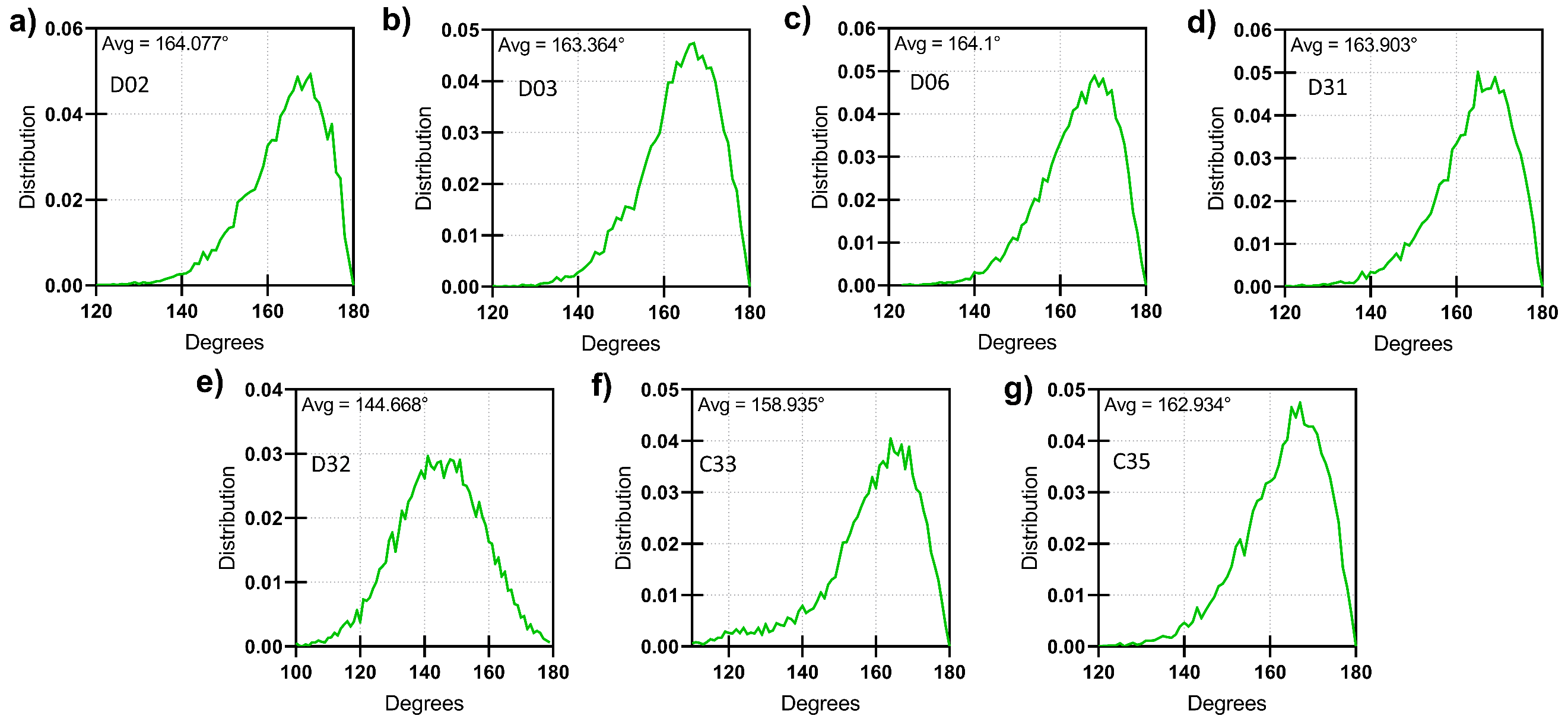
**

**Supplementary Figure-S8. Angular distribution of the H-bond interaction between the residue M156 and compounds a) D02, b) D03, c) D06, d) D31, e) D32, f) D33, and g) D35 during the MD simulation**

**
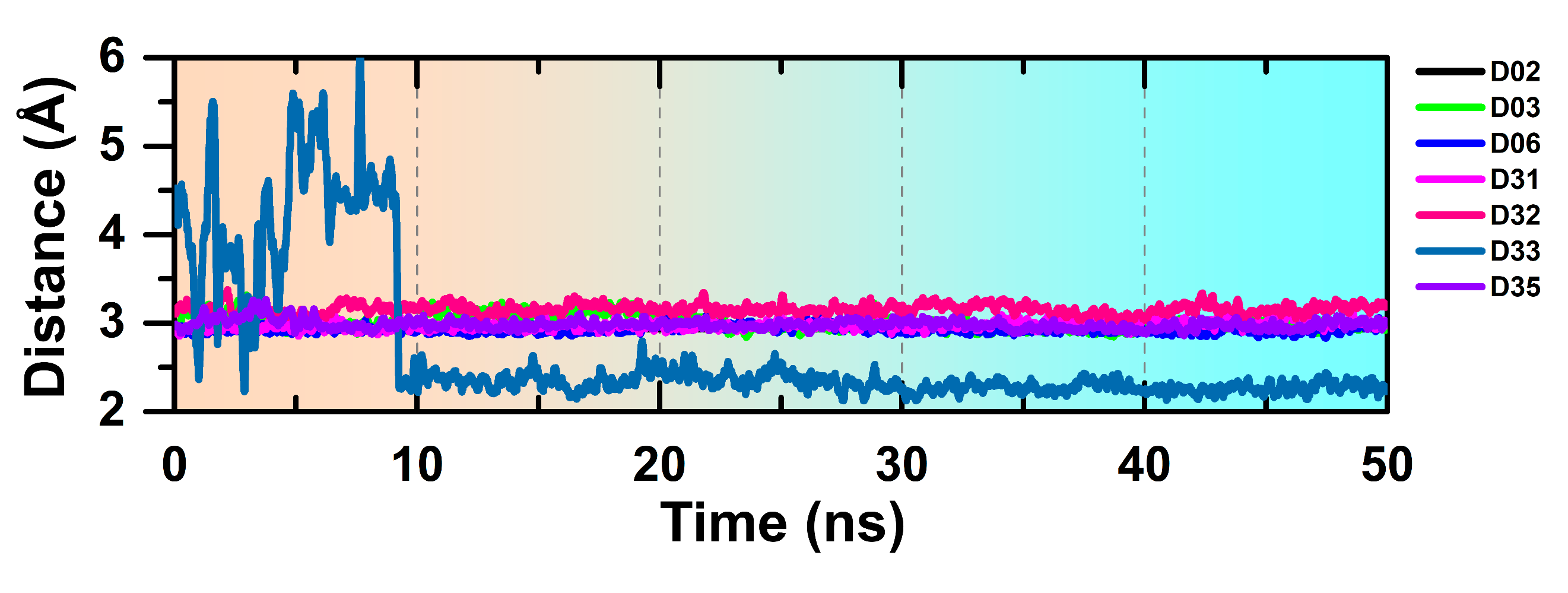
**

**Supplementary Figure-S9. Distance of the H-bond interaction between the residue M156 and compounds a) D02, b) D03, c) D06, d) D31, e) D32, f) D33, and g) D35 during the production simulation**

**Supplementary Table-S8. Average H-bond distance and H-bond angle formation with residue M156**

| **#Cpd.** | **Avg. H-bond distance from residue M156 in Å** | **Avg. H-bond angle with residue M165 (D-H-A)** |
| --- | --- | --- |
| **C03** | 2.9 ± 0.01 | 163.47° |
| **C08** | 2.9 ± 0.01 | 164.69° |
| **C11** | 2.9 ± 0.01 | 161.63° |
| **C13** | 3.0 ± 0.01 | 164.23° |
| **C19** | 3.3 ± 0.02 | 151.91° |
| **C22** | 4.9 ± 0.02 | 144.90° |
| **C25** | 2.9 ± 0.01 | 164.52° |
| **C29** | 2.9 ± 0.01 | 159.68° |
| **C34** | 2.9 ± 0.01 | 161.89° |
| **C37** | 2.9 ± 0.01 | 162.26° |
| **D02** | 2.9 ± 0.01 | 164.07° |
| **D03** | 3.0 ± 0.01 | 163.36° |
| **D06** | 2.9 ± 0.01 | 164.10° |
| **D31** | 2.9 ± 0.01 | 163.90° |
| **D32** | 3.1 ± 0.61 | 144.66° |
| **D33** | 2.6 ± 0.08 | 158.93° |
| **D35** | 2.9 ± 0.01 | 162.93° |

**#Cpd:** Compounds**; D-H-A:** Donor-Hydrogen-Acceptor**; Avg.:** Average

**Supplementary Table-S9.** **Residue based Binding energy contribution from MMPBSA to the designed compounds in kJ/mol**

| **Residue** | **D02** | **D03** | **D06** | **D31** | **D32** | **D33** | **D35** |
| --- | --- | --- | --- | --- | --- | --- | --- |
| **E89** | -9.18 | -7.0612 | -7.93 | -5.81 | -6.40 | -6.45 | -4.76 |
| **V90** | -9.07 | -9.1735 | -7.11 | -8.61 | -8.24 | -7.81 | -9.06 |
| **K105** | 34.69 | 44.2439 | 42.03 | 16.83 | 27.16 | 20.76 | 31.57 |
| **E124** | -11.03 | -12.4689 | -13.88 | -9.61 | -8.64 | -9.99 | -7.17 |
| **R125** | 2.32 | 2.2394 | 3.11 | 1.91 | 1.74 | 2.54 | 1.14 |
| **M153** | -2.90 | -3.7989 | -3.84 | -1.19 | -3.92 | -0.84 | -2.68 |
| **Y155** | -3.94 | -4.2575 | -6.22 | -4.23 | -3.75 | -2.93 | -3.81 |
| **M156** | -4.34 | -4.5787 | -6.50 | -3.40 | -2.76 | -3.50 | -3.86 |
| **K200** | 4.64 | 3.1422 | 6.54 | 5.29 | 6.82 | 5.89 | 0.63 |
| **D202** | -5.58 | -2.9276 | 4.47 | -11.52 | -6.27 | -11.76 | -3.23 |
| **L205** | -5.41 | -5.8829 | -5.83 | -4.47 | -5.37 | -3.83 | -4.52 |
| **D207** | -3.26 | -2.3531 | -3.29 | -3.03 | -3.06 | -2.70 | -1.17 |
| **D216** | -4.59 | 8.3028 | -4.73 | -6.52 | 2.56 | -12.63 | 3.25 |
| **D364** | -3.00 | -2.3625 | -2.70 | -1.87 | -1.51 | -3.19 | -1.42 |
| **F368** | -3.75 | -3.2654 | -1.51 | -5.36 | -5.93 | -5.14 | -2.27 |
| **D369** | -7.19 | -4.7898 | -5.38 | -2.06 | -4.75 | -4.16 | -2.55 |
| **D370** | -4.57 | -2.9621 | -2.98 | -2.97 | -3.24 | -3.21 | -1.77 |
